# Terminamines K–S, Antimetastatic Pregnane Alkaloids from the Whole Herb of *Pachysandra terminalis*

**DOI:** 10.3390/molecules21101283

**Published:** 2016-09-26

**Authors:** Xiang-Yu Li, Yang Yu, Miao Jia, Mei-Na Jin, Nan Qin, Chuan Zhao, Hong-Quan Duan

**Affiliations:** Tianjin Key Laboratory on Technologies Enabling Development Clinical Therapeutics and Diagnostics (Theranostics), School of Pharmacy, Research Center of Basic Medical Sciences, Tianjin Medical University, Tianjin 300070, China; lovelxybeibei@163.com (X.-Y.L.); yuyang@tmu.edu.cn (Y.Y.); ijiamiao4ev@126.com (M.J.); jinmeina@tmu.edu.cn (M.-N.J.); nanqin.qn@163.com (N.Q.)

**Keywords:** *Pachysandra terminalis*, Buxaceae, pregnane alkaloids, anti-metastatic activity, structure-activity relationships

## Abstract

Nine new pregnane alkaloids (**1**–**9**), together with eight known alkaloids (**10**–**17**), were isolated from the whole herb of *Pachysandra terminalis*. Their structures were elucidated on the basis of spectroscopic analyses. In addition, the isolates were examined for their ability to inhibit the migration of MDA-MB-231 cells induced by the chemokine epidermal growth factor (EGF). Alkaloids **1**, **5**, **7**, **9**, **12**, and **17** presented significant anti-metastasis activities compared with the positive reagent, LY294002.

## 1. Introduction

Steroidal alkaloids are an important class of secondary metabolites that occur in plants and also in certain higher animals and marine invertebrates, and are the major constituents in plants of Apocynaceae, Buxaceae, Liliaceae, and Solanaceae [1,2]. *Pachysandra terminalis* Sieb. et Zucc. (Buxaceae) is distributed in the southwestern region of China and Japan. It has been used as a traditional medicine against pain and stomachache, with pregnane alkaloids as its main bioactive components [3]. Previous pharmacological research has shown that these compounds produce a wide range of pharmacological effects, such as antiulcer [4], cytotoxic [3], anti-leishmanial [5], anti-cancer [6], anti-breast cancer metastatic activities [7,8], as well as estrogen biosynthesis-promoting effects [9]. As part of an ongoing research program to identify bioactive steroidal alkaloids with anti-metastatic effects nine new (**1**–**9**) and eight known (**10**–**17**) pregnane alkaloids were isolated from the whole herb of *Pachysandra terminalis*. The isolation, structure elucidation, and anti-metastatic effects of these compounds are described, and their structure-activity relationships is discussed.

## 2. Results and Discussion

### 2.1. Purification and Characterization

**Figure 1:**
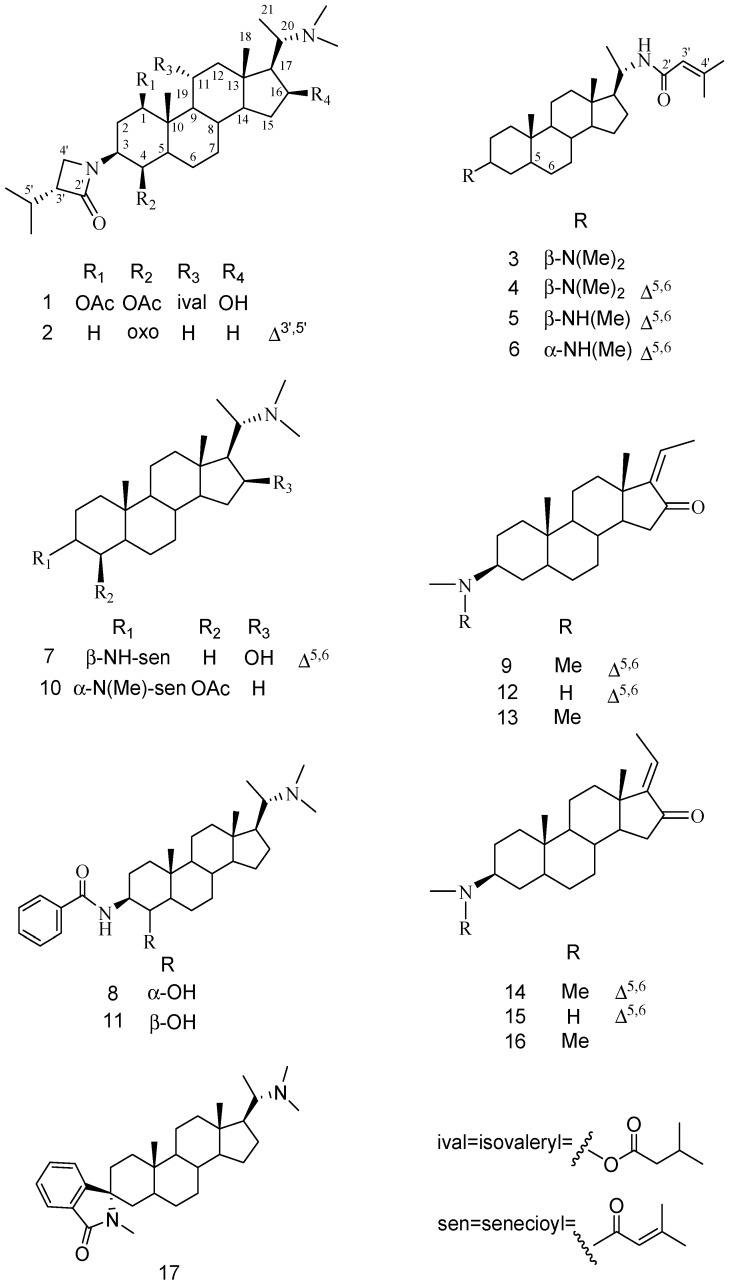
Structures of compounds **1**–**17**.

The total alkaloid extract from the whole plants of *P. terminalis* was separated by silica gel column chromatography and semi-preparative reversed phase HPLC to yield nine new pregnane alkaloids, named terminamines K–S (**1**–**9**), and eight known alkaloids. By comparison of their spectroscopic data with values available in the literature, the known alkaloids were identified as: (+)-pachysandrine B (**10**) [10], epipachysandrine-A (**11**) [11], 3β-methylamino 16-oxo 5,17(20) *cis*-pregnadiene (**12**) [12], Z-salignone (**13**) [13], terminamine H (**14**) [8], 3β-methylamino 16-oxo 5,17(20) trans-pregnadiene (**15**) [12], E-salignone (**16**) [13], and (+)-spiropachysine (**17**) [14] (Figure 1). Structures of **1**–**9** were determined based on the 1D- and 2D-NMR, and mass spectrometric analytical results.

### 2.2. Structural Elucidation of Compounds ***1**–**9***

Alkaloid **1** was obtained as a yellow oil and its molecular formula was established as C_38_H_62_N_2_O_8_ on the basis of the positive-ion mode HRESIMS data (*m/z* 675.4617 [M + H]^+^, calcd for C_38_H_63_N_2_O_8_, 675.4584). The IR spectrum displayed absorption bands at 3362 and 1750 cm^−1^, accounting for the presence of hydroxyl and carbonyl groups. The ^1^H-NMR spectrum of **1** revealed four oxygenated protons [δ_H_ 5.05 (1H, m), 4.95 (1H, dd, *J* = 11.2, 4.8 Hz), 4.55 (1H, dd, *J* = 12.0, 4.0 Hz), 4.33 (1H, m)], four tertiary methyls [δ_H_ 2.05, 1.94, 1.15, 0.86 (each 3H, s)], five secondary methyls [δ_H_ 1.03, 1.07 (each 3H, d, *J* = 6.7 Hz); 0.89, 1.04 (each 3H, d, *J* = 7.6 Hz); 0.89 (3H, d, *J* = 6.4 Hz)], and six N(CH_3_)_2_ protons [δ_H_ 2.23 (6H, s)]. The ^1^H-NMR spectrum supported a steroidal skeleton in **1**, with two methyl singlets resonating upfield at δ_H_ 0.86 and 1.15, characteristic of C-18 and C-19 methyls, respectively [15].

**Figure 2:**
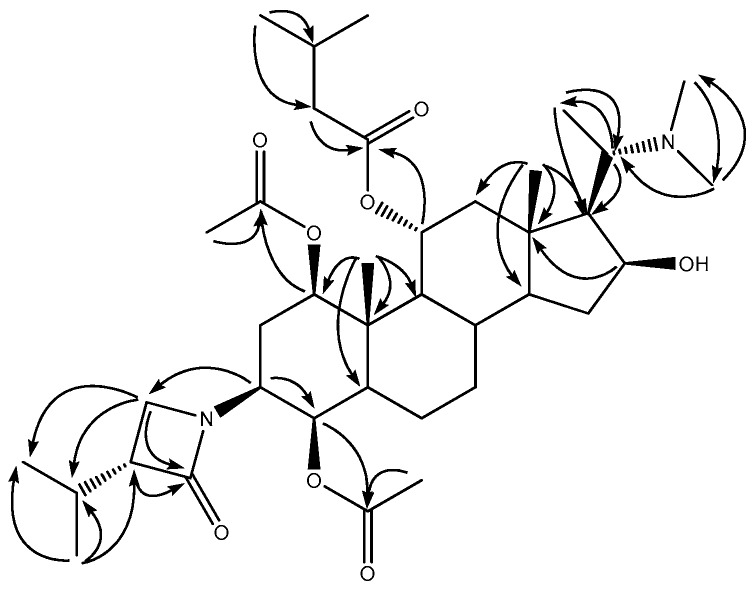
Key HMBC (H → C) correlations for **1**.

In combination with analysis of the ^1^H-^1^H COSY, HSQC, and HMBC spectra, an isopropyl lactam group (δ_C_ 170.1, 56.2, 45.4, 27.5, 19.8, 19.7), one isovaleryl group (δ_C_ 176.3, 26.5, 40.2, 14.1, 10.8), together with two acetyl groups (δ_C_ 170.8 × 2, 21.9, 21.0) were identified. The remaining 21 carbon signals were assigned to the pregnane skeleton, similar to those of terminamine C [7]. By comparison of the NMR spectroscopic data with **10**, **1** was proposed to have an acetyl group at the C-4 position, instead of a hydroxyl group. In the HMBC spectrum, correlations were observed for the resonance at δ_H_ 4.55 (H-4) with the signals at δ_C_ 170.8 (acetyl group), 48.0 (C-3), 44.2 (C-5), and 22.9 (C-6). Thus, the acetyl group was assigned at C-4 (Figure 2).

**Figure 3:**
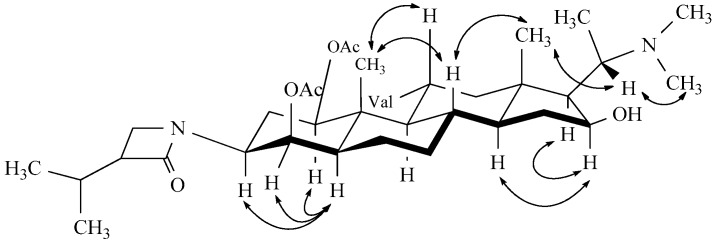
Key NOEs for **1**.

In the ROESY spectrum, the proton signal of H-5 (1.70) correlated with H-1 (4.95), H-3 (4.18), and H-4 (4.55); the signal H-16 (4.33) correlated with H-14 (0.99) and H-17 (1.21); H-11 (5.05) with H-19 (1.15); and H-20 (2.89) with H-18 (0.86). The above observations indicated α-orientations for H-1, H-3, H-4, H-16, and H-17, and a β-orientation for H-11 (Figure 3). The above 3′α-isopropyl)-lactam-11α-ival-5α-pregnane, and named terminamine K (Figure 1).

Terminamine L (**2**) was isolated as a white powder, the HRESIMS showed a molecular ion peak at *m*/*z* 455.3631 ([M + H]^+^, calcd for C_29_H_47_N_2_O_2_, 455.3638). The ^1^H-NMR spectrum of **2** featured four tertiary methyl signals at δ_H_ 2.04, 1.70, 0.75, and 0.65 (each 3H, s), a secondary methyl at δ_H_ 0.90 (3H, d, *J* = 6.4 Hz), and six N(CH_3_)_2_ protons at δ_H_ 2.21 (6H, s). The ^13^C-NMR spectroscopic data for **2** were similar to those of terminamine E [7], except for the signals of C-4 (Table 1), and **2** was deduced to be the 4-oxo derivative of terminamine E. In the HMBC spectrum, two methyl groups (δ_H_ 2.04 and 1.70) showed a correlation with double bond carbons (δ_C_ 130.9 and 135.1), while the proton signals at δ_H_ 3.93 and 3.68 (H-4′) showed correlations with δ_C_ 130.9 (C=C) and δ_C_ 165.1 (C=O), suggesting a 3′-isopropylidene lactam. On the other hand, the proton signals at δ_H_ 4.42 (H-3) correlated with signals at δ_C_ 27.6 (C-2), 207.6 (C-4), 45.8, and 165.1 (β-lactam moiety). Thus, the β-lactam and ketone groups were assigned to C-3 and C-4, respectively. Finally, all of the available data identified the structure of **2** as 20α-dimethylamino-3β-(3′-isopropylidene)-lactam-5α-pregn-4-one (Figure 1).

Terminamine M (**3**) was obtained as a white powder and assigned the molecular formula C_28_H_48_N_2_O from its HRESIMS. The ^1^H-NMR spectrum of **3** revealed the presence of two proton signals [δ_H_ 5.12 (1H, d, *J* = 9.1 Hz), 4.05 (1H, m)] connected with electron-withdrawing groups, an olefinic proton [δ_H_ 5.49 (1H, s)], four tertiary methyls [δ_H_ 2.13, 1.82, 0.77, and 0.72 (each 3H, s)], a secondary methyl [δ_H_ 1.16 (3H, d, *J* = 6.3 Hz)], and two NCH_3_ methyls [δ_H_ 2.36 (6H, s)].

The ^13^C-NMR and DEPT spectra of **3** indicated the presence of seven methyls (δ_C_ 41.3, 41.3, 27.1, 21.9, 19.7, 12.4, 12.3), a double bond (δ_C_ 150.0, 119.0), and one ketone carbonyl (δ_C_ 165.8). In addition, nine methylenes, seven methines, and two quaternary carbons were also observed. Analysis of the ^1^H- and ^13^C-NMR data supported a pregnane skeleton in **3** (Table 2), and resembled those of epipachysamine E [7,10]. However, the signals of the related positions of *N*,*N*-dimethyl and senecioylamino groups {δ_H_ 2.47 (H-3), 4.05 (H-20), 2.36 [H-N(Me)_2_]; δ_C_ 64.4 (C-3), 47.2 (C-20), 41.3 [C-N(Me)_2_]} in **3** showed a significant difference with those of epipachysamine E {δ_H_ 3.42 (H-3), 3.79 (H-20), 2.17 [H-N(Me)_2_]; δ_C_ 61.1 (C-3), 48.5 (C-20), 35.5 [C-N(Me)_2_]}.

In the HMBC spectrum, the methyl proton signals at δ_H_ 2.36 (NMe_2_) correlated with the carbon signal at δ_C_ 64.4 (C-3), suggesting the *N*,*N*-dimethyl group was located at C-3. On the other hand, the proton signal at δ_H_ 4.05 (H-20) correlated with the proton signal at δ_H_ 5.12 (H-NH) in the COSY spectrum, while the proton signal at δ_H_ 5.12 (H-NH) correlated with the proton signals at δ_H_ 5.49 (H-3′) and 1.16 (H-21) in the ROESY spectrum, which indicated that the senecioylamino group was assigned to position C-20. Furthermore, the ROESY correlations between H-18 (δ_H_ 0.72) and H-20 (δ_H_ 4.05) indicated a β-orientation for H-17. However, there was no further chemical or spectral evidence for the identification of the relative configuration for C-3, except for the 1D-NMR data [8,13]. A series of 3-isomers of pregnane alkaloid derivatives were synthesized using the stereoselective Mitsunobu reaction [16] by this group [17,18,19]. The results suggested that the C-3 signal of 3β-dimethylamino derivatives appeared more downfield. The carbon signal at δ_C_ 64.4 (C-3) was in correspondence to that of the 3β-dimethylamino derivatives. Thus, the structure of **3** was assigned as 3β-dimethylamino-20α-senecioylamino-5α-pregnane (Figure 1).

A molecular formula of C_28_H_46_N_2_O was determined from the HRESIMS data for terminamine N (**4**). The ^1^H- and ^13^C-NMR spectroscopic data of **4** were similar to those of **3**, except for the presence of a C=C bond (Table 2). Thus, **4** was deduced to be the 5,6-dehydro derivative of **3**. In the HMBC spectrum, the correlations from Me-19 (δ_H_ 0.98) to C-5 (δ_C_ 142.0); H-6 (δ_H_ 5.35) to C-4 (δ_C_ 35.2), C-7 (δ_C_ 31.9), C-8 (δ_C_ 31.7), and C-10 (δ_C_ 36.9) disclosed that the C=C bond was located at the C-5-C-6 position. Consequently, the structure of **4** was elucidated as 3β-dimethylamino-20α-senecioylamino-pregn-5-ene (Figure 1).

Terminamines O (**5**) and P (**6**) had the same molecular formula, C_27_H_44_N_2_O, on the basis of HRESIMS data. A comparison of the in NMR spectroscopic data with those of **4** indicated that **5** and **6** resembled **4**, except for a missing N-methyl group. By comparing the 1D NMR spectra, it was considered that alkaloids **5** and **6** were stereoisomers at C-3 (Table 2). Compared with literature reports [20], together with the 3-isomers of the pregnane alkaloid derivatives [17,18,19], the β-orientation of the 3-NH(Me) group in **5** could be assigned due to the chemical shift of C-3 (δ_C_ 59.5), while the upfield shift of C-3 (δ_C_ 54.9) leads to the assignment of a 3α-NH(Me) group in **6**. Further analysis of the HSQC, HMBC, and ROESY NMR data was used to establish the structure of compounds **5** and **6** as 3β-methylamino-20α-senecioylamino-pregn-5-ene, and 3α-methylamino-20α-senecioylamino-pregn-5-ene, respectively (Figure 1).

Terminamine Q (**7**) had a molecular formula of C_28_H_46_N_2_O_2_ by HRESIMS. Comparison of the ^1^H- and ^13^C-NMR data (Table 3) showed that compound **7** has the same pregnane skeleton as epipachysamine E [3], and was thought to be the 5,6-dehydro-16-hydroxy derivative of epipachysamine E. In the HMBC spectrum, the proton signal at δ_H_ 1.00 (H-19) correlated with the carbon signals at δ_C_ 37.9 (C-1), 140.3 (C-5), 50.1 (C-9), and 36.6 (C-10); the signal at δ_H_ 5.38 (H-6) correlated with the carbon signals at δ_C_ 39.4 (C-4), 31.9 (C-7), 31.3 (C-8), and 36.6 (C-10); while the signal at δ_H_ 0.89 (H_3_-18) correlated with the carbon signals at δ_C_ 41.4 (C-13), 53.6 (C-14), and 58.9 (C-17). In addition, the proton signal at δ_H_ 4.36 (H-16) correlated with the signal at δ_H_ 1.26 (H-17) in the COSY spectrum. The above observations indicated that the C=C double bond was located at C-5-C-6, and the hydroxyl group was assigned to C-16. Furthermore, the ROESY correlations between H-16 (δ_H_ 4.36) and H-17 (δ_H_ 1.26), and H-20 (δ_H_ 2.97) and H_3_-18 (δ_H_ 0.89) indicated that the relative configuration of the hydroxyl group was 16β. As a result, the structure of **7** was elucidated as 20α-dimethylamino-16β-hydroxy-3β-senecioylamino-pregn-5-ene.

Terminamine R (**8**) had a same molecular formula of C_30_H_46_N_2_O_2_ as epipachysandrine A (**11**), as evidenced by a molecular ion peak at *m*/*z* 467.3638 [M + H]^+^ from its HRESIMS spectrum. The ^1^H- and ^13^C-NMR spectroscopic data (Table 3) of **8** were similar to those of **11** [20], and its structure was considered to be the 4-isomer of **11**. The α-orientation of the 4-OH group was determined by the ROESY correlation of H-4 (δ_H_ 3.78) with H-19 (δ_H_ 0.88). Thus, the structure of **8** was determined as 3β-benzoylamino-4α-hydroxy-5α-pregnane.

A molecular formula of C_23_H_35_NO was determined from the HRESIMS data (*m*/*z* 342.2822) for terminamine S (**9**). From the comparison of the ^1^H- and ^13^C-NMR of **9** (Table 3) with those of terminamine H [8], alkaloid **9** was identified to be the 17(20)*Z*-derivative of **14** due to the upfield signal at H-20 (δ_H_ 5.73 in **9**, 6.52 in **14**). The β-orientation of the 3-dimethylamino group was determined as the same manner as described for the identification of alkaloids **3**–**6**. Thus, the structure of **9** was determined as (*Z*)-3β-(dimethylamino)-17(20)-pregn-5-en-16-one.

**Figure 4:**
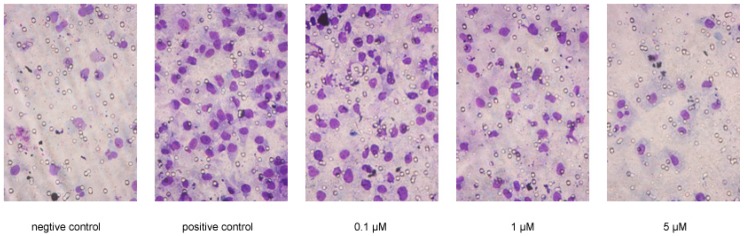
Anti-invasive effect of compound 1 on MDA-MB-231 cells by chemotaxis assay.

Compounds **1**–**17** were tested for their inhibitory effects on the chemotaxis of the breast adenocarcinoma cell line MDA-MB-231 induced by epidermal growth factor (EGF). All compounds were tested using non-cytotoxic concentrations. Compared with the positive control LY294002 (IC_50_ = 1.18), compounds **1**, **5**, **7**, **9**, **12**, and **17** presented significant anti-metastatic activities with IC_50_ values of 0.58, 0.71, 1.38, 1.01, 1.91, and 0.31 μM, respectively (Table 4, Figure 4).

Since the methylamino or dimethylamino groups are common moieties among the active pregnane alkaloids, these substituted groups are necessary for the activity of these compounds. In addition, *Z*-configuration of C-17(20) could improve anti-metastatic activity compared with isomers **9**, **12**–**16**.

## 3. Materials and Methods 

### 3.1. General

Optical rotation was measured with a Jasco P-2000 polarimeter (Tokyo, Japan). UV spectra were recorded in methanol on a Hitachi U-3310 UV-vis spectrophotometer (Tokyo, Japan). IR spectra were acquired using a Nicolet 380 FT-IR spectrophotometer (Waltham, MA, USA). The NMR spectra were obtained using a Bruker AVANCE III 400 instrument (400 MHz for ^1^H-NMR and 100 MHz for ^13^C-NMR; Bruker Biospin AG, Fällanden, Switzerland) using TMS as an internal standard. ^1^H-^1^H COSY, HSQC, HMBC, and ROESY NMR experiments were also performed on the Bruker AVANCE III 400 instrument, using standard pulse sequences. The high-resolution mass spectra were recorded on a Varian 7.0 T ESI-MS (Palo Alto, CA, USA). HPLC was performed using a JASCO Gulliver Series (Tokyo, Japan) with PU-2089 pump, RI-2031, and UV-2075 detector. Semi-preparative HPLC column chromatography was carried out on YMC-Pack Polymer-C18 (6 μm, 10 mm × 200 mm), YMC-pack SIL-06 (YMC Co., Ltd., Kyoto, Japan). Open column chromatography was performed using silica gel (Qingdao Haiyang Chemical Co., Ltd., Qingdao, China). The chemotaxis chambers and membranes were purchased from Neuroprobe (Gaithersburg, MD, USA) and human EGF was obtained from Peprotech (Rocky Hill, NJ, USA).

### 3.2. Plant Materials

Entire plants of *Pachysandra terminalis* were collected in April 2011 from Hefeng, Hubei Province, China. The plants were identified by D.R. Wan (School of Life Sciences, South Central University for Nationalities, Wuhan, Hubei Province, China) and a voucher specimen (D20110903) was deposited at the School of Pharmacy, Tianjin Medical University, Tianjin, China.

### 3.3. Extraction and Isolation

The dried plant material (7.6 kg) was extracted with 75% ethanol (40 L × 3, 6 h each time) under reflux. The resultant extract was concentrated in vacuo to a gummy residue (1750 g), suspended in water, and partitioned with petroleum ether to defat. The water-soluble fraction was adjusted to pH 1 with 2% HCl and then centrifuged under 5400 *g* for 10 min. The acid-soluble fraction was alkalinized to pH 11 with NH_3_∙H_2_O followed by exhaustive extraction with EtOAc. The EtOAc extract (32.0 g) was chromatographed on a silica gel column (300–400 mesh, 10 × 120 cm, 1000 g) and eluted using a solvent gradient system (petroleum ether–EtOAc–Et_2_NH, 20:1:1, 20:1.2:1, 20:2:1, 20:3.5:1, 20:5:1, 20:10:1, *v*/*v*/*v*) to produce eight fractions (A–H) on the basis of TLC analysis. Fraction B (1.59 g) was separated by semi-preparative HPLC (polymer C-18, MeOH–H_2_O–Et_2_NH, 50:50:1.5, *v*/*v*/*v*) to produce seven fractions (B_1_–B_7_). Fraction B_2_ (22.0 mg) was purified by semi-preparative HPLC (polymer C-18) using MeOH–H_2_O–Et_2_NH (50:50:1.5, *v*/*v*/*v*) as mobile phase to give **5** (9.4 mg). Fraction B_4_ (305.1 mg) provided **8** (11.9 mg) and **11** (16.2 mg) after purification over semi-preparative HPLC (polymer C-18, MeOH–H_2_O–Et_2_NH, 80:20:1.5, *v*/*v*/*v*). Fraction D (1.3 g) was applied to a silica gel column with mixtures of petroleum ether–EtOAc–Et_2_NH (20:1.5:1, 20:2:1, 20:2.5:1, 20:4:1, *v*/*v*/*v*) as the eluent to yield 10 fractions (D_1_–D_10_). Alkaloid **6** (21.6 mg) was obtained from fraction D_7_ (68.3 mg) separated on semi-prep. HPLC (polymer C-18) with the mobile phase of MeOH–H_2_O–Et_2_NH (80:20:1.5, *v*/*v*/*v*). Alkaloids **1** (67.4 mg) and **17** (5.0 mg) were obtained by a silica gel column using a stepwise gradient-elution with mixtures of petroleum ether–EtOAc–Et_2_NH (from 20:0.5:1, to 20:1.5:1, *v*/*v*/*v*) from D_3_ (305.2 mg) and D_5_ (88.4 mg), respectively. Fraction E (1.53 g) was chromatographed on semi-preparative HPLC (SIL, petroleum ether–EtOAc–Et_2_NH, 20:10:1) to produce **2** (3.0 mg), **10** (47.2 mg), and 11 fractions (E_1_–E_11_). Fraction E_8_ (325.6 mg) was subjected to semi-preparative HPLC (SIL) and eluted with petroleum ether–EtOAc–Et_2_NH (20:10:1) to obtain **9** (13.5 mg), **13** (36.3 mg), **14** (28.7 mg), and **16** (8.3 mg). Fraction G (1.76 g) was chromatographed by semi-preparative HPLC (SIL, petroleum ether–EtOAc–Et_2_NH, 40:60:5) to obtain **7** (352.6 mg) and nine fractions (G_1_–G_9_). Compound **4** (2.0 mg) was obtained from fraction G_7_ (90.2 mg) by semi-preparative HPLC (SIL) with petroleum ether–EtOAc–Et_2_NH (20:10:1). Using the same method, **3** (10.3 mg), **12** (17.8 mg), and **15** (13.1 mg) were separated from G_9_ (175.9 mg).

Terminamine K (**1**). Yellow oil; [α]${}_{D}^{20}$ −36.3 (*c* 1.13, CHCl_3_); IR (KBr) ν_max_: 3362, 2963, 2873, 1750, 1461, 1371 cm^−1^; For ^1^H- (CDCl_3_, 400 MHz) and ^13^C-NMR (CDCl_3_, 100 MHz) spectroscopic data, see Table 1; HRESIMS: *m*/*z* 675.4617 [M + H]^+^ (calcd for C_38_H_63_N_2_O_8_, 675.4584). For details of the NMR spectrum, please see the Supplementary Materials Figures S1–S6.

Terminamine L (**2**). White powder; [α]${}_{D}^{20}$ +52.4 (*c* 0.21, CHCl_3_); IR (KBr) ν_max_: 3388, 2934, 1736, 1385 cm^−1^; For ^1^H- (CDCl_3_, 400 MHz) and ^13^C-NMR (CDCl_3_, 100 MHz) spectroscopic data, see Table 1; HRESIMS: *m*/*z* 455.3631 [M + H]^+^ (calcd for C_29_H_47_N_2_O_2_, 455.3638). For details of the NMR spectrum, please see the Supplementary Materials Figures S7–S12.

Terminamine M (**3**). White powder; [α]${}_{D}^{20}$ +21.4 (*c* 0.14, CHCl_3_); UV (MeOH) λ_max_ (log ε) 218 (3.32) nm; IR (KBr) ν_max_: 3305, 2930, 2866, 2772, 1631, 1533 cm^−1^;For ^1^H- (CDCl_3_, 400 MHz) and ^13^C-NMR (CDCl_3_, 100 MHz) spectroscopic data, see Table 2; HRESIMS: *m*/*z* 429.3846 [M + H]^+^ (calcd for C_28_H_49_N_2_O, 429.3845). For details of the NMR spectrum, please see the Supplementary Materials Figures S13–S18.

Terminamine N (**4**). Yellow oil; [α]${}_{D}^{20}$ −36.4 (*c* 0.22, CHCl_3_); UV (MeOH) λ_max_ (log ε) 214 (3.46) nm; IR (KBr) ν_max_: 2967, 2931, 2868, 2775, 1668, 1628 cm^−1^; For ^1^H- (CDCl_3_, 400 MHz) and ^13^C-NMR (CDCl_3_, 100 MHz) spectroscopic data, see Table 2; HRESIMS: *m*/*z* 427.3700 [M + H]^+^ (calcd for C_28_H_47_N_2_O, 427.3688). For details of the NMR spectrum, please see the Supplementary Materials Figures S19–S24.

Terminamine O (**5**). Yellow powder; [α]${}_{D}^{20}$ −36.2 (*c* 0.58, CHCl_3_); UV (MeOH) λ_max_ (log ε) 216 (3.55) nm; IR (KBr) ν_max_: 3296, 2934, 2869, 2788, 1718, 1668, 1630, 1535, 1452, 1378, 1261, 1178 cm^−1^; For ^1^H- (CDCl_3_, 400 MHz) and ^13^C-NMR (CDCl_3_, 100 MHz) spectroscopic data, see Table 2; HRESIMS: *m*/*z* 413.3517 [M + H]^+^ (calcd for C_27_H_45_N_2_O, 413.3532). For details of the NMR spectrum, please see the Supplementary Materials Figures S25–S30.

Terminamine P (**6**). Yellow powder; [α]${}_{D}^{20}$ −57.1 (*c* 1.05, CHCl_3_); UV (MeOH) λ_max_ (log ε) 215 (3.64) nm; IR (KBr) ν_max_: 3295, 2955, 2925, 2853, 1461, 1377 cm^−1^; For ^1^H- (CDCl_3_, 400 MHz) and ^13^C-NMR (CDCl_3_, 100 MHz) spectroscopic data, see Table 2; HRESIMS: *m*/*z* 413.3549 [M + H]^+^ (calcd for C_27_H_45_N_2_O, 413.3532). For details of the NMR spectrum, please see the Supplementary Materials Figures S31–S36.

Terminamine Q (**7**). Yellow oil; [α]${}_{D}^{20}$ −46.7 (*c* 0.30, CHCl_3_); UV (MeOH) λ_max_ (log ε) 240 (3.72) nm; IR (KBr) ν_max_: 3272, 2937, 2870, 1741, 1629, 1447, 1253 cm^−1^; For ^1^H- (CDCl_3_, 400 MHz) and ^13^C-NMR (CDCl_3_, 100 MHz) spectroscopic data, see Table 3; HRESIMS: *m*/*z* 443.3661 [M + H]^+^ (calcd for C_28_H_47_N_2_O_2_, 443.3638). For details of the NMR spectrum, please see the Supplementary Materials Figures S37–S42.

Terminamine R (**8**). Colorless oil; [α]${}_{D}^{20}$ +27.8 (*c* 0.18, CHCl_3_); UV (MeOH) λ_max_ (log ε) 220 (3.55) nm; IR (KBr) ν_max_: 3339, 2930, 1649, 1524, 1471, 1383 cm^−1^; For ^1^H- (CDCl_3_, 400 MHz) and ^13^C-NMR (CDCl_3_, 100 MHz) spectroscopic data, see Table 3; HRESIMS: *m*/*z* 467.3638 [M + H]^+^ (calcd for C_30_H_47_N_2_O_2_, 467.3638). For details of the NMR spectrum, please see the Supplementary Materials Figures S43–S48.

Terminamine S (**9**). Yellow amorphous powder; [α]${}_{D}^{20}$ −38.7 (*c* 0.31, CHCl_3_); UV (MeOH) λ_max_ (log ε) 240 (2.66) nm; IR (KBr) ν_max_: 2969, 2933, 2853, 2774, 1711, 1458 cm^−1^; For ^1^H- (CDCl_3_, 400 MHz) and ^13^C-NMR (CDCl_3_, 100 MHz) spectroscopic data, see Table 3; HRESIMS: *m*/*z* 342.2822 [M + H]^+^ (calcd for C_23_H_36_NO, 342.2797). For details of the NMR spectrum, please see the Supplementary Materials Figures S49–S54.

### 3.4. Chemotaxis Assay

The chemotaxis invasion assay was performed as previously described [21], using non-toxic concentrations of each compound, at which the inhibition rate on breast cancer cell growth was below 20%. MDA-MB-231 cells were pretreated with the alkaloids at the indicated concentrations for 24 h at 37 °C in six-well cell culture plates, then resuspended in binding medium [RPMI 1640 (Roswell Park Memorial Institute, Biological Industries, Kibbutz Beit Haemek, Israel), containing 0.1% bovine serum albumin (BSA) and 25 mM 4-2-hydroxyethyl-1-piperazineethanesulfonic acid] at a density of 0.5 × 10^6^ cells/mL and placed into the upper chamber (50 μL/well). A chemoattractant (EGF; 1 ng/mL, 30 μL/well) was loaded into the lower chemotaxis chamber. The 8-μm filter membranes (Neuroprobe), which had been pretreated with 0.001% fibronectin in serum-free medium at 4 °C overnight and air-dried, were inserted between the upper and lower chambers. The cells were incubated at 37 °C in 5% CO_2_ for 3.5 h; then the whole chemotaxis chamber was inverted and the lower chamber was removed. The filter membranes were held using two small clamps and the upper side of the membrane was scraped lightly three to five times with rinsing between scrapes. The membrane was then fixed and stained with a three-step stain set (Richard-Allan Scientific, Kalamazoo, MI, USA, recorder No.: 3300). The number of migrating cells in three separate fields was counted by light microscopy at 400×. Cells of sample group were pretreated with alkaloids and were induced with EGF. Positive control cells got EGF induction but without alkaloids pretreatment. Negative control cells got no alkaloid pretreatment or EGF induction. The inhibitory ratio (IR) was calculated as follows:

IR% = (1 − number of invasive cells of sample group/number of invasive cells of positive control group) × 100%
(1)

The potencies of the products were expressed as the median inhibitory concentration (IC_50_) values. LY294002 (BioSource International, Camarillo, CA, USA) was used as a positive reagent for this assay [22,23].

## 4. Conclusions

In summary, this work described the isolation and structure identification of nine new (**1**–**9**) and eight known (**10**–**17**) pregnane alkaloids from the whole plant of *Pachysandra terminalis*. Alkaloids **1**, **5**, **7**, **9**, **12**, and **17** presented significant anti-metastasis activities compared with the positive reagent, LY294002. In addition, the methylamino or dimethylamino groups are necessary for the activity of these compounds, and *Z*-configuration of C-17(20) could improve the anti-metastatic activity.

## Figures and Tables

**Table 1:** NMR Spectroscopic data (400 MHz, CDCl_3_) for Terminamine K (**1**) and Terminamine L (**2**).

Position	Terminamine K (1)		Terminamine L (2)	
	δ_C_	δ_H_ (*J* in Hz)	δ_C_	δ_H_ (*J* in Hz)
1	74.6	4.95, dd (11.2, 4.8)	36.5	1.96, m
				1.54, m
2	28.1	1.70, m	27.6	1.93, m
		2.33, m		1.55, m
3	48.0	4.18, m	58.5	4.42, m
4	72.2	4.55, dd (12.0, 4.0)	207.6	-
5	44.2	1.70, m	58.2	2.16, m
6	22.9	1.33, m	20.3	1.62, m
		1.82, m		1.44, m
7	32.1	1.00, m	30.2	1.75, m
		1.88, m		0.81, m
8	34.1	1.69, m	34.9	1.30, m
9	56.6	1.41, m	54.2	0.96, m
10	42.2	-	42.5	-
11	71.4	5.05, m	21.6	1.55, m
				1.26, m
12	45.0	2.60, m	39.6	1.91, m
		0.80, m		1.20, m
13	41.2		41.7	
14	51.4	0.99, m	54.7	1.40, m
15	35.1	2.21, m	24.0	1.62, m
		1.19, m		1.08, m
16	72.5	4.33, m	27.5	1.92, m
17	58.8	1.21, m	56.1	1.09, m
18	14.4	0.86, s	12.3	0.65, s
19	10.6	1.15, s	13.8	0.75, s
20	56.6	2.89, m	61.3	2.54, m
21	9.8	0.89, d (6.4)	10.0	0.90, d (6.4)
N(Me)_2_	39.8	2.23, s	39.9	2.21, s
2′	170.1	-	165.1	-
3′	56.2	3.06, m	130.9	-
4′	45.4	3.70, m	45.8	3.93, d (6.5)
		3.18, m		3.68, d (6.5)
5′	27.5	2.06, m	135.1	-
5′-(Me)_2_	19.8	1.03, d (6.7)	19.9	2.04, s
	19.7	1.07, d (6.7)	20.3	1.70, s
1-OAc	21.9	1.94, s		
	170.8			
4-OAc	21.0	2.05, s		
	170.8			
11-Val	176.3			
	26.5	1.43, m		
		1.65, m		
	40.2	2.31, m		
	14.1	1.04, d (7.6)		
	10.8	0.89, d (7.6)		

**Table 2:** NMR Spectroscopic data (400 MHz, CDCl_3_) for Terminamine M-P (**3**–**6**).

Position	Terminamine M (3)		Terminamine N (4)		Terminamine O (5)		Terminamine P (6)	
	δ_C_	δ_H_ (*J* in Hz)	δ_C_	δ_H_ (*J* in Hz)	δ_C_	δ_H_ (*J* in Hz)	δ_C_	δ_H_ (*J* in Hz)
1	37.6	1.79, m	38.3	1.90, m	37.6	1.90, m	33.3	1.30, m
		0.96, m		1.06, m		1.06, m		1.60, m
2	24.0	1.57, m	24.0	1.60, m	24.0	1.60, m	25.0	1.74, m
		1.09, m		1.13, m		1.11, m		
3	64.4	2.47, m	64.9	2.23, m	59.5	2.59, m	54.9	2.81, m
4	32.0	0.87, m	35.2	2.24, m	37.3	2.35, m	36.4	2.48, m
		1.67, m						2.12, m
5	45.6	1.09, m	142.0	-	140.0	-	139.0	-
6	30.5	1.55, m	120.7	5.35, m	121.9	5.37, m	122.9	5.35, m
		1.32, m						
7	28.9	1.28, m	31.9	1.99, m	31.8	1.99, m	31.8	1.95, m
				1.53, m		1.50, m		1.58, m
8	35.3	1.37, m	31.7	1.49, m	31.7	1.47, m	31.7	1.47, m
9	54.3	0.64, m	50.2	0.95, m	50.1	0.95, m	50.0	1.09, m
10	35.7	-	36.9	-	37.0	-	37.2	-
11	21.0	1.51, m	20.8	1.49, m	20.8	1.48, m	20.6	1.48, m
		1.27, m						
12	39.4	1.91, m	39.2	1.96, m	39.1	1.95, m	39.1	1.94, m
		1.11, m		1.16, m		1.17, m		1.16, m
13	42.2	-	41.9	-	41.9	-	41.9	-
14	56.6	1.02, m	56.8	1.07, m	56.8	1.03, m	56.7	1.05, m
15	24.1	1.82, m	25.0	1.81, m	24.0	1.59, m	24.0	1.59, m
		1.47, m		1.50, m		1.11, m		1.10, m
16	26.8	1.75, m	26.8	1.78, m	26.7	1.77, m	26.8	1.77, m
		1.46, m		1.49, m		1.49, m		1.47, m
17	56.9	1.28, m	56.9	1.31, m	56.8	1.31, m	56.7	1.31, m
18	12.3	0.72, s	12.2	0.75, s	12.2	0.75, s	12.1	0.75, s
19	12.4	0.77, s	19.4	0.98, s	19.3	1.00, s	19.1	1.02, s
20	47.2	4.05, m	47.2	4.06, m	47.2	4.05, m	47.2	4.05, m
21	21.9	1.16, d (6.3)	21.9	1.17, d (6.8)	21.9	1.17, d (6.4)	21.9	1.17, d (6.5)
N(Me)_2_	41.3	2.36, s	41.6	2.35, s	31.7	2.49, s	33.1	2.38, s
NH	-	5.12, d (9.1)	-	5.13, d (8.8)	-	5.13, d (8.8)	-	5.21, d (8.1)
2′	165.8	-	165.8	-	165.8	-	165.8	-
3′	119.0	5.49, s	119.0	5.49, s	119.0	5.49, s	119.1	5.50, s
4′	150.0	-	150.0	-	150.0	-	149.9	-
4′-(Me)_2_	19.7	2.13, s	19.7	2.14, s	19.7	2.14, s	19.7	2.14, s
	27.1	1.82, s	27.1	1.82, s	27.1	1.82, s	27.1	1.82, s

**Table 3:** NMR Spectroscopic data (400 MHz, CDCl_3_) for Terminamine Q-S (**7**–**9**).

Position	Terminamine Q (7)		Terminamine R (8)		Terminamine S (9)	
	δ_C_	δ_H_ (*J* in Hz)	δ_C_	δ_H_ (*J* in Hz)	δ_C_	δ_H_ (*J* in Hz)
1	37.9	1.84, m	32.8	1.60, m	35.6	1.88, m
		1.17, m		1.10, m		1.38, m
2	29.3	1.87, m	27.1	2.15, m	20.6	1.72, m
		1.35, m		1.89, m		1.62, m
3	49.3	3.73, m	51.2	4.47, m	65.1	2.15, m
4	39.4	2.34, m	71.1	3.78, m	32.0	1.63, m
		2.09, m				
5	140.3	-	47.4	1.22, m	142.3	-
6	121.7	5.38, m	29.7	1.26, m	120.2	5.34, br d (4.8)
7	31.9	2.02, m	31.2	1.78, m	31.7	1.99, m
		1.54, m		0.89, m		
8	31.3	1.57, m	34.5	1.41, m	30.9	1.65, m
9	50.1	0.98, m	54.4	0.77, m	49.8	1.40, m
10	36.6	-	37.2	-	37.1	-
11	20.7	1.47, m	20.7	1.54, m	20.5	1.72, m
				1.29, m		1.61, m
12	40.0	1.84, m	39.7	1.90, m	35.2	2.23, m
		1.13, m		1.20, m		
13	41.4	-	42.5	-	43.0	-
14	53.6	0.92, m	56.1	1.08, m	50.8	1.46, m
15	34.9	2.18, m	24.3	1.76, m	39.5	2.20, m
		1.25, m		1.25, m	39.5	2.03, m
16	72.6	4.36, m	24.9	1.92, m	208.7	-
17	58.9	1.26, m	52.5	1.47, m	148.2	-
18	14.1	0.89, s	12.3	0.69, s	19.4	0.94, s
19	19.4	1.00, s	13.0	0.88, s	19.4	1.03, s
20	56.8	2.97, m	64.0	3.21, m	130.2	5.73, q (7.2)
21	9.9	0.95, d (6.4)	12.7	1.32, d (6.0)	14.0	2.09, d (7.2)
N(Me)_2_	39.9	2.25, s	39.4	2.48, s	41.7	2.31, s
NH	-	5.20, d (8.0)	-	6.38, d (6.7)		
2′	166.2	-	170	-		
3′	118.8	5.52, s	134.4	-		
4′	150.4	-	127.1	7.80, d (7.2)		
5′			128.6	7.46, m		
6′			131.7	7.53, m		
7′			128.6	7.46, m		
8′			127.1	7.80, d (7.2)		
4′-(Me)_2_	19.7	2.15, s				
	27.1	1.83, s				

**Table 4:** Inhibitory Effects of Compounds **1**−**17** ^a^ on the Invasion of MDA-MB-231 Cells.

Compound	IC_50_^a^ (μM)	Compound	IC_50_^a^ (μM)
**1**	0.58 ± 0.06	**10**	2.35 ± 0.31
**2**	4.65 ± 0.82	**11**	5.71 ± 0.84
**3**	3.14 ± 0.51	**12**	1.91 ± 0.15
**4**	8.12 ± 0.75	**13**	3.32 ± 0.28
**5**	0.71 ± 0.06	**14**	2.16 ± 0.23
**6**	6.47 ± 0.54	**15**	3.98 ± 0.36
**7**	1.38 ± 0.17	**16**	6.91 ± 0.74
**8**	8.62 ± 1.03	**17**	0.31 ± 0.01
**9**	1.01 ± 0.09		
LY294002 ^b^	1.18 ± 0.14		

^a^ The values are means ± SD (*n* = 5); ^b^ LY294002 as a positive reagent.

## Supplementary Materials

Supplementary materials can be accessed at: http://www.mdpi.com/1420-3049/21/10/1283/s1.
