# Supplementary material for: Terminamines K–S, Antimetastatic Pregnane Alkaloids from the Whole Herb of Pachysandra terminalis
Source: Molecules. 2016 Sep 26;21(10):1283. doi: 10.3390/molecules21101283 (PMC6273090; doi:10.3390/molecules21101283)
Supplement: Supplementary file 1 [file molecules-21-01283-s001.pdf]

Supplementary Material: Terminamines K–S, Antimetastatic Pregnane Alkaloids from the Whole Herb of *Pachysandra terminalis*

Xiang-Yu Li, Yang Yu, Miao Jia, Mei-Na Jin, Nan Qin, Chuan Zhao and Hong-Quan Duan

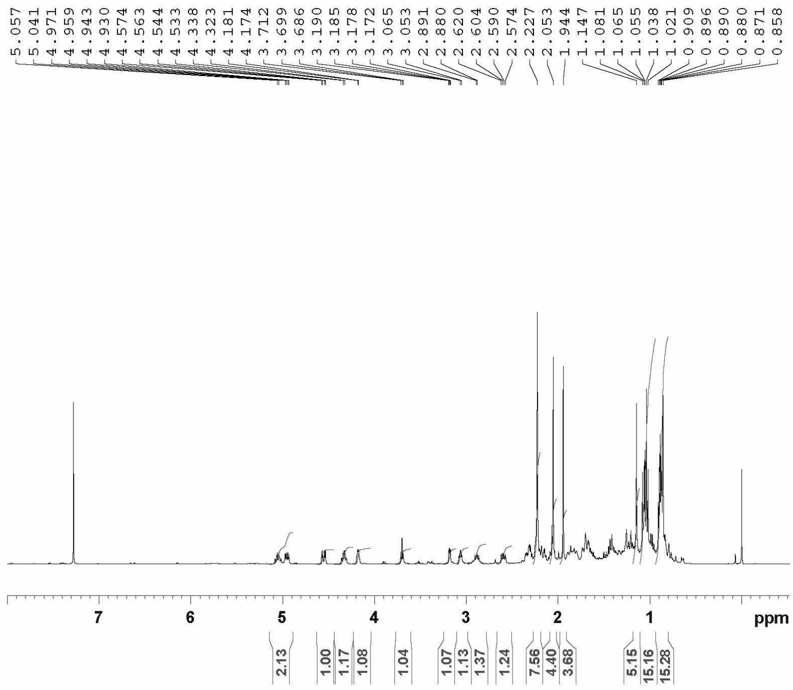

Figure S1. ^{1}H-NMR spectrum of compound **1**.

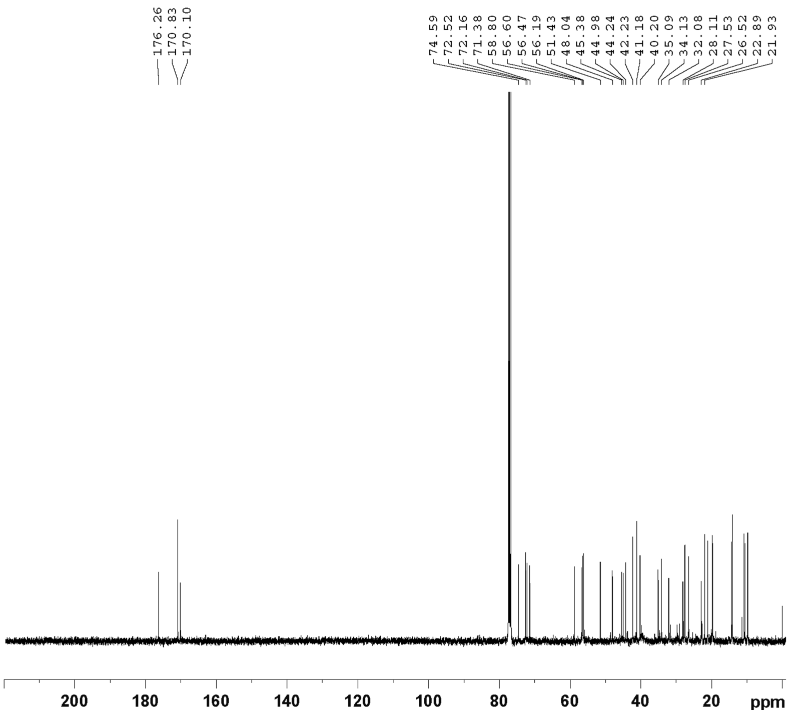

Figure S2. ^{13}C-NMR spectrum of compound **1**.

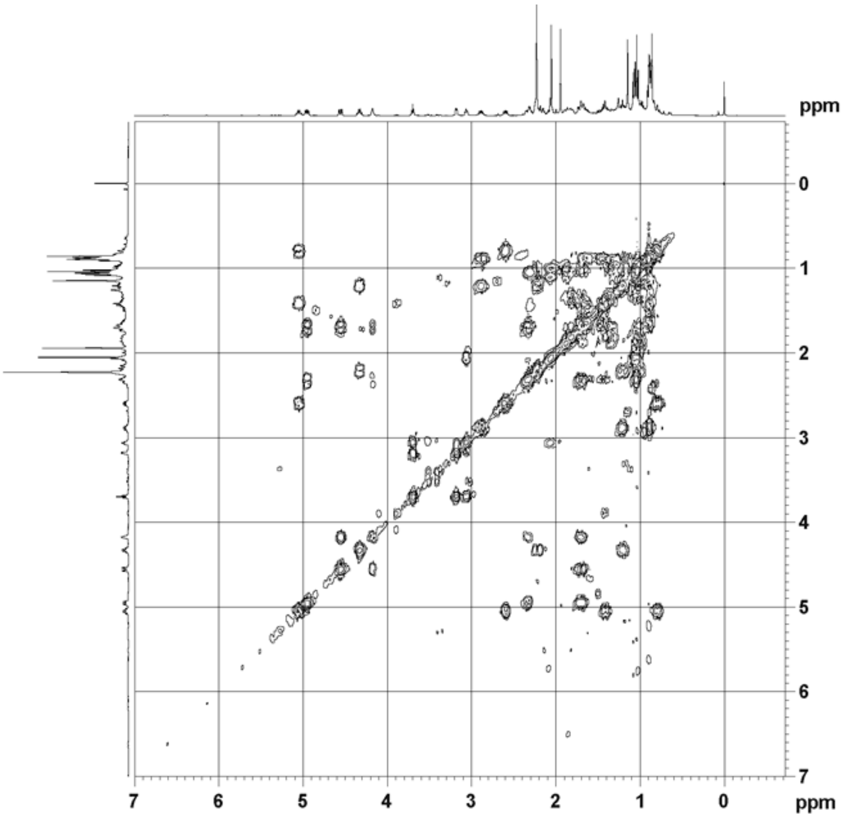

Figure S3. ^{1}H-^{1}H COSY spectrum of compound **1**.

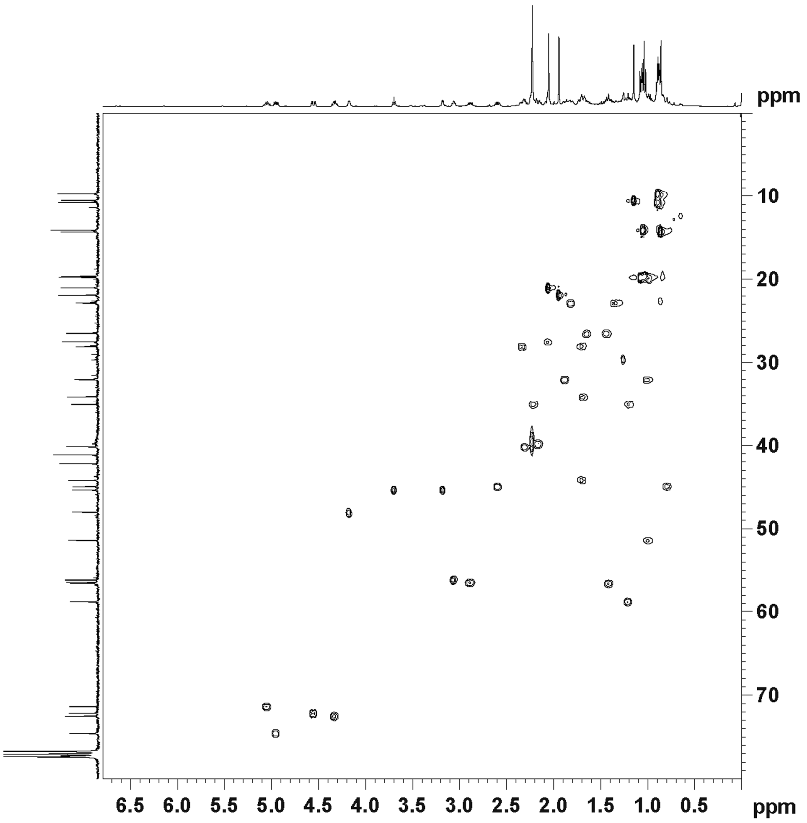

Figure S4. HSQC spectrum of compound **1**.

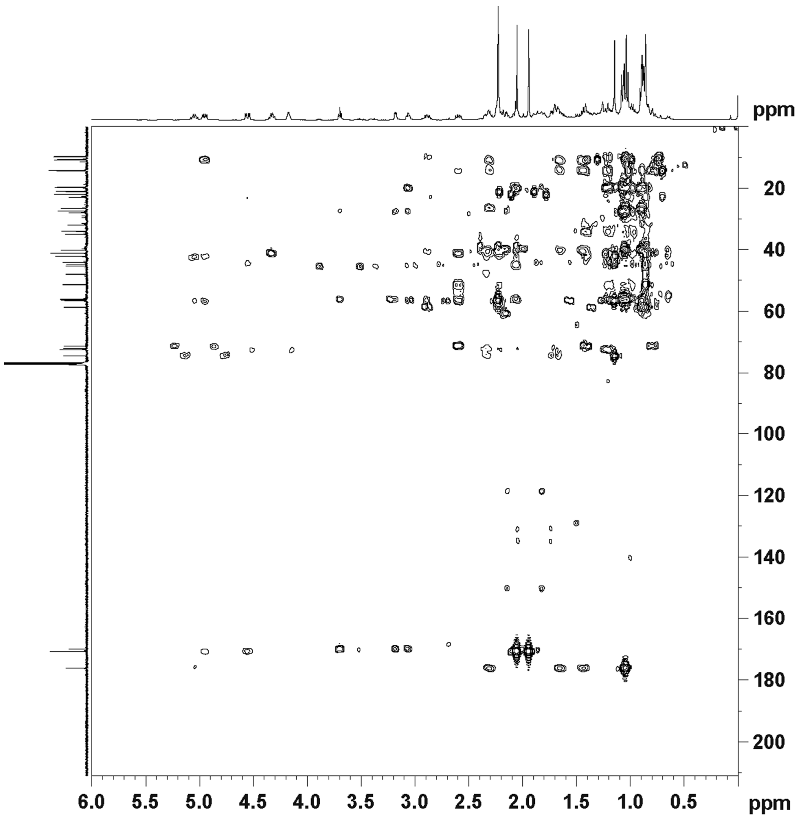

Figure S5. HMBC spectrum of compound **1**.

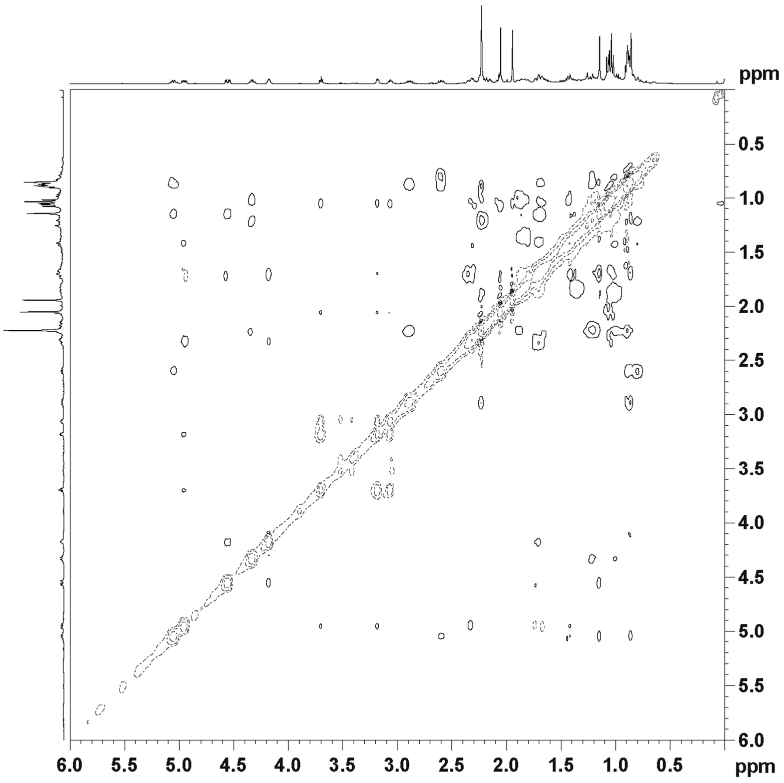

Figure S6. ROESY spectrum of compound **1**.

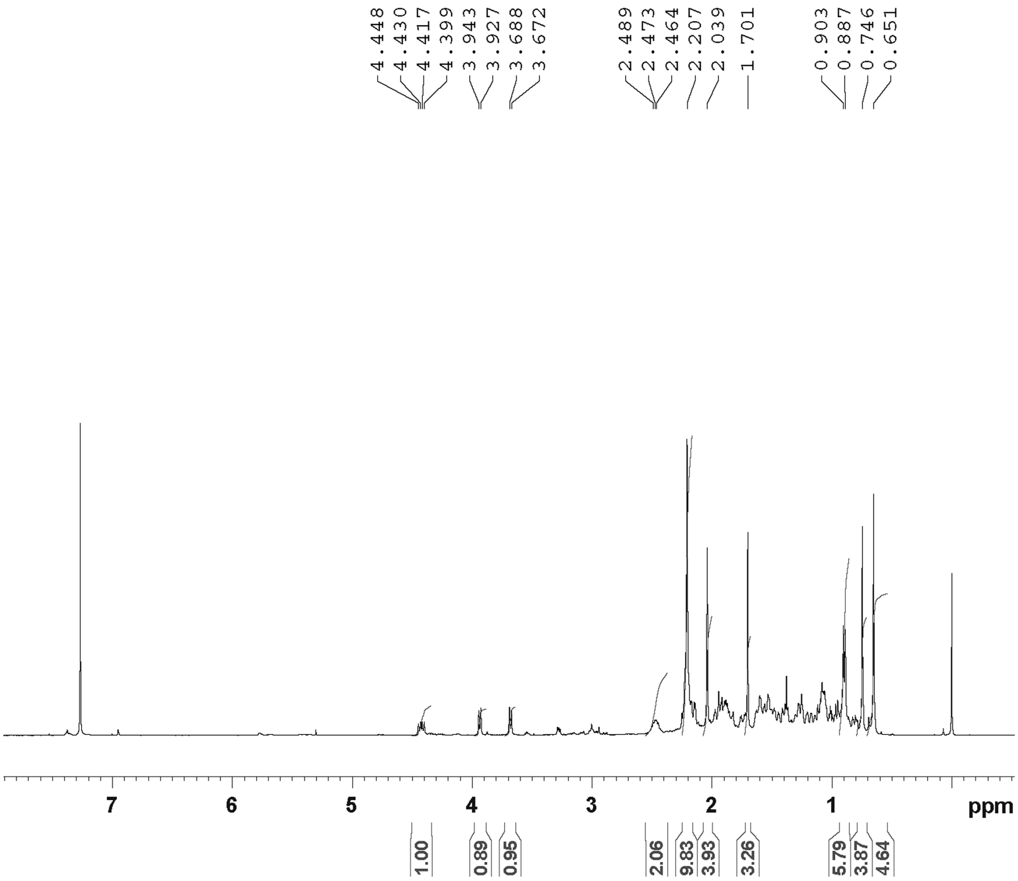

Figure S7. ^{1}H-NMR spectrum of compound **2**.

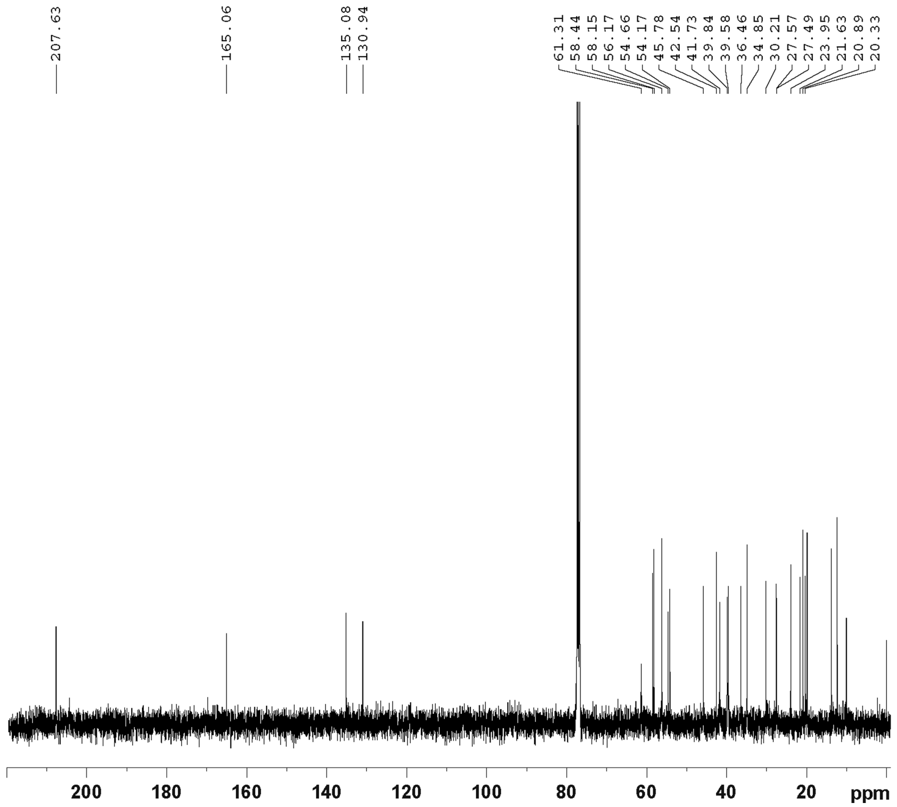

Figure S8. ^{13}C-NMR spectrum of compound **2**.

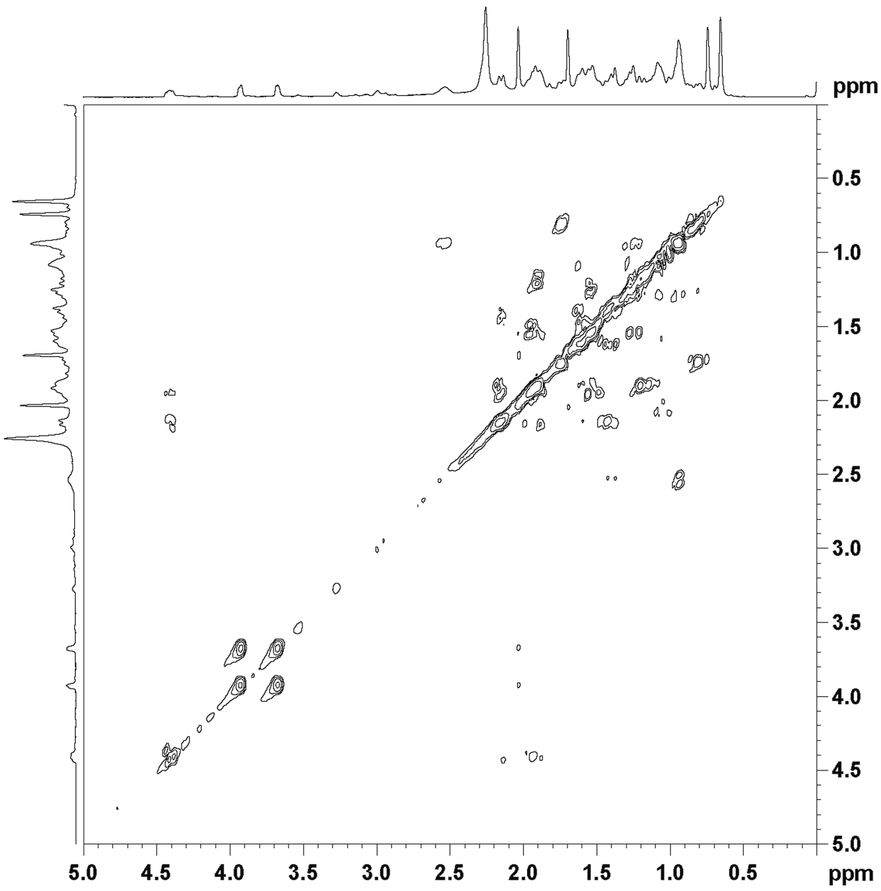

Figure S9. ^{1}H-^{1}H COSY spectrum of compound **2**.

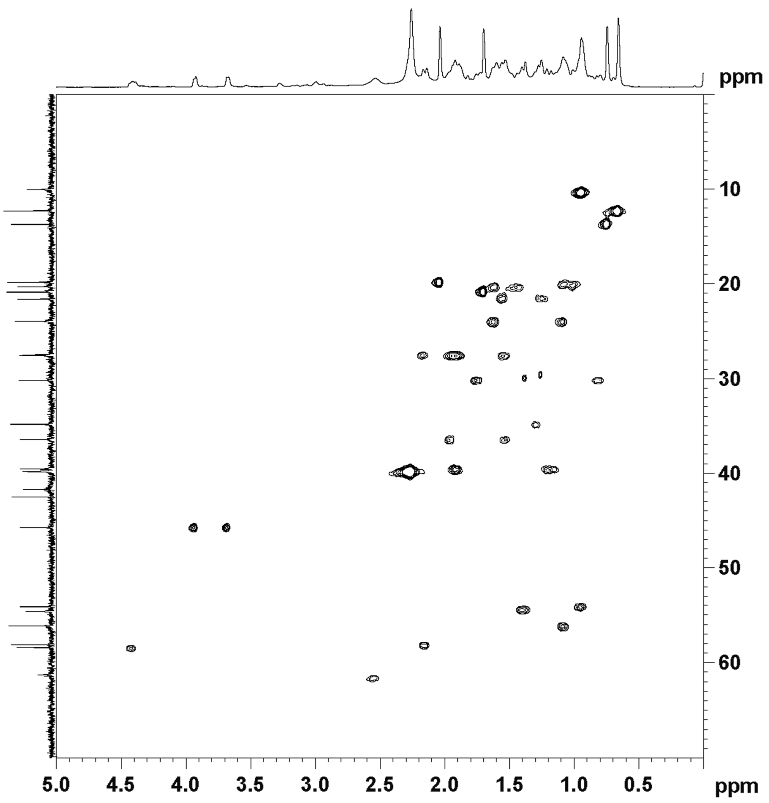

Figure S10. HSQC spectrum of compound **2**.

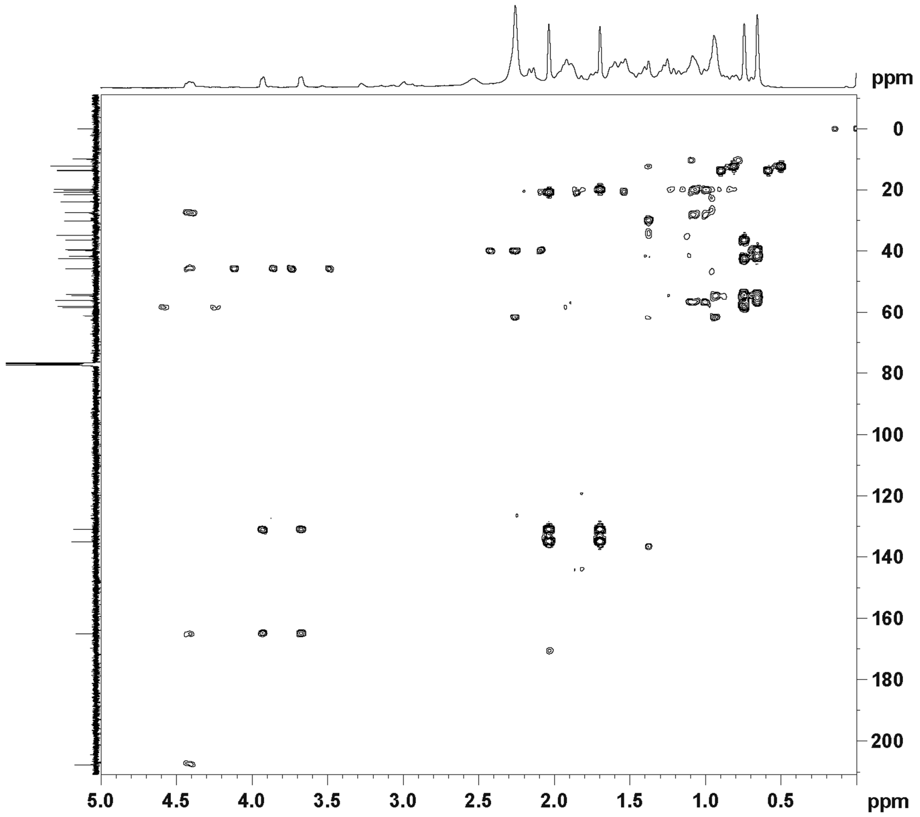

Figure S11. HMBC spectrum of compound **2**.

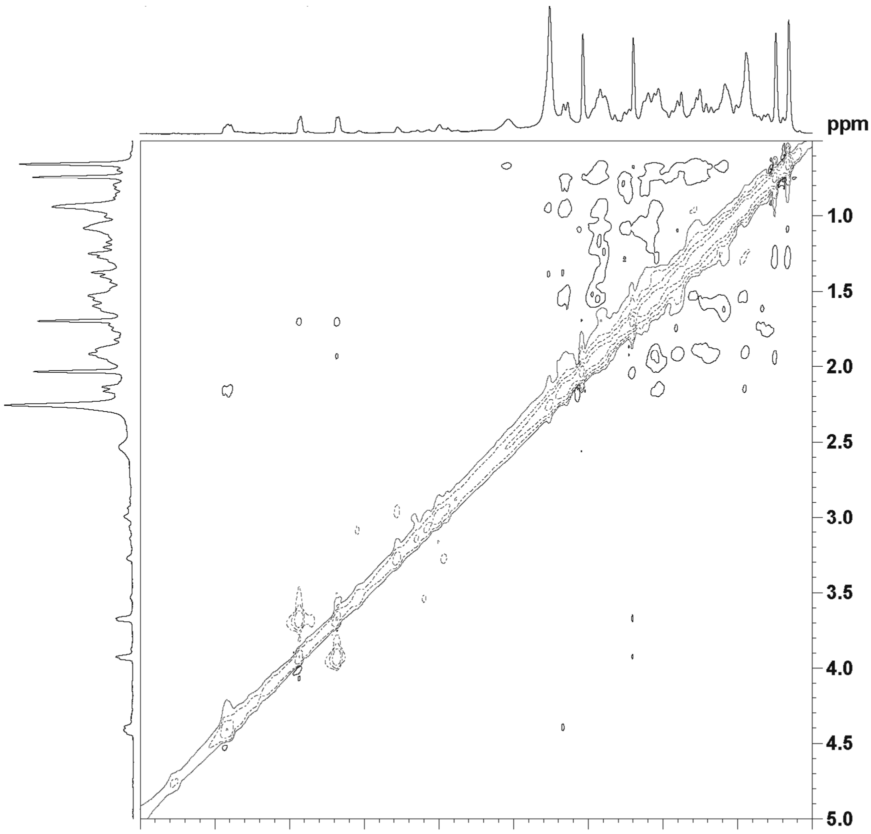

Figure S12. ROESY spectrum of compound **2**.

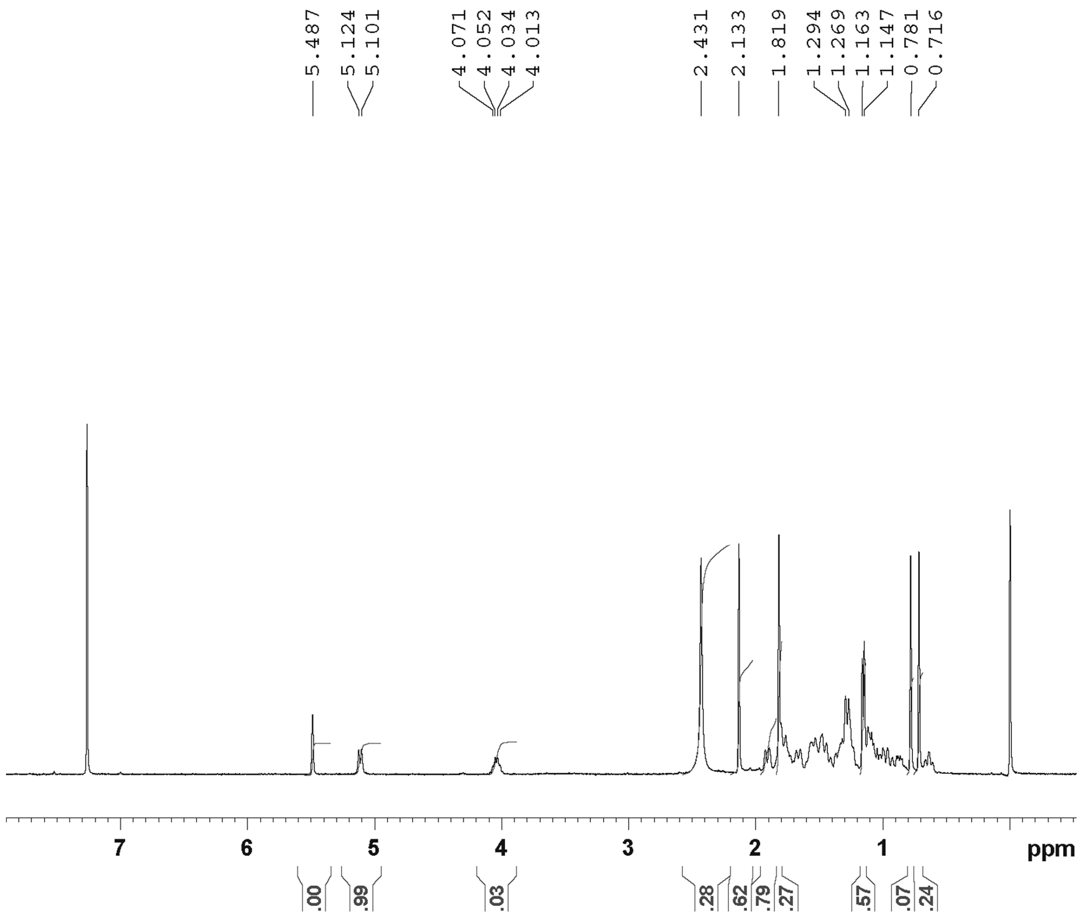

Figure S13. ¹H-NMR spectrum of compound **3**.

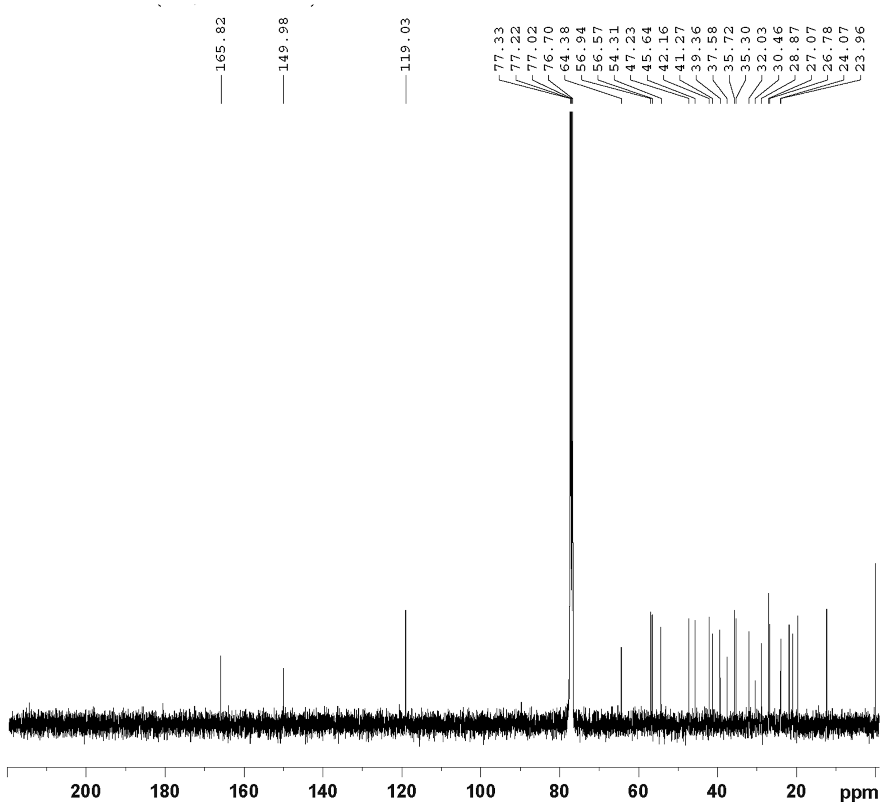

Figure S14. ¹³C-NMR spectrum of compound **3**.

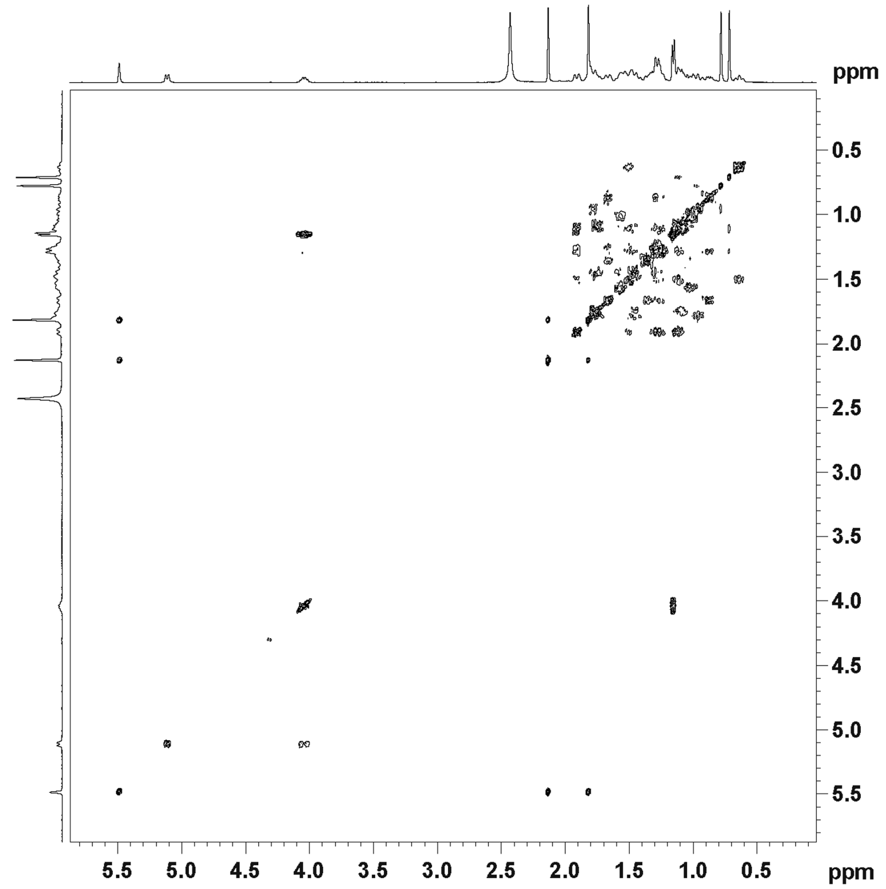

Figure S15. ^{1}H-^{1}H COSY spectrum of compound **3**.

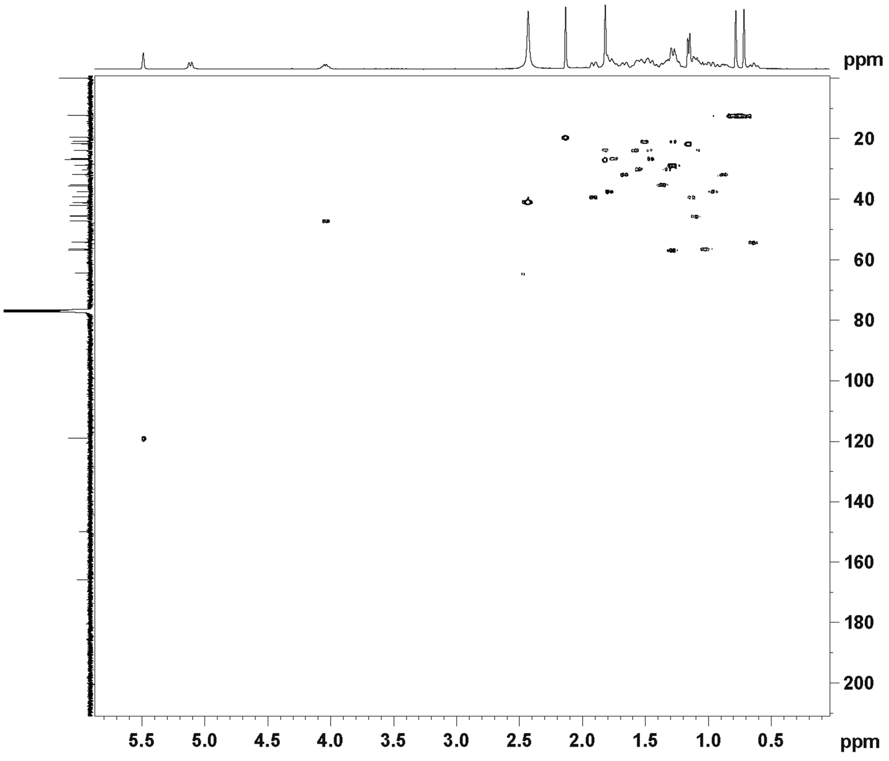

Figure S16. HSQC spectrum of compound **3**.

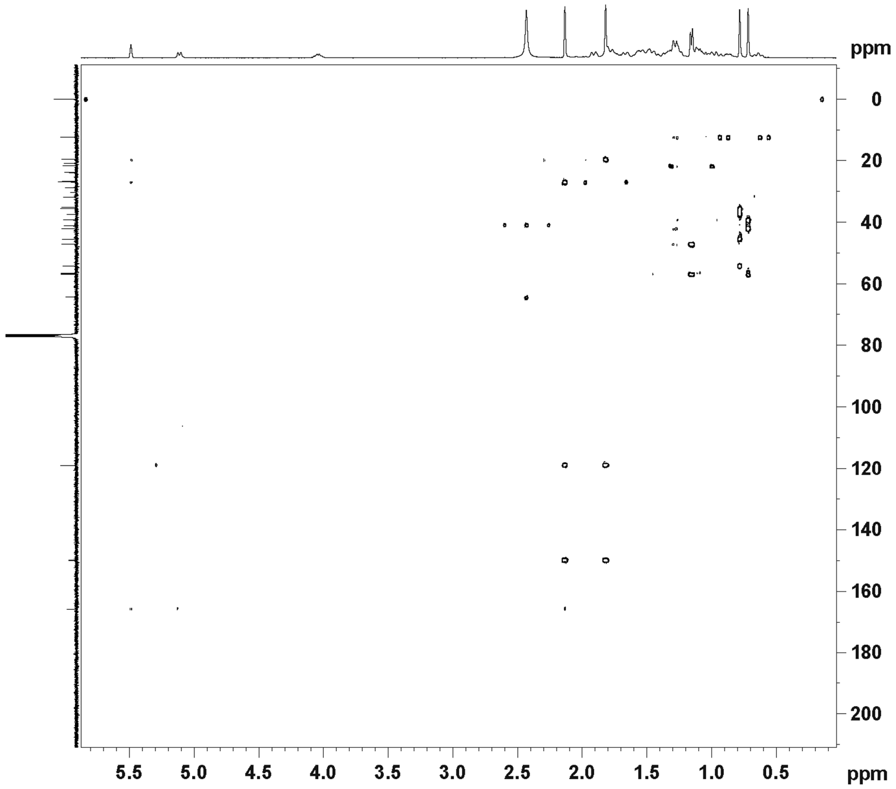

Figure S17. HMBC spectrum of compound **3**.

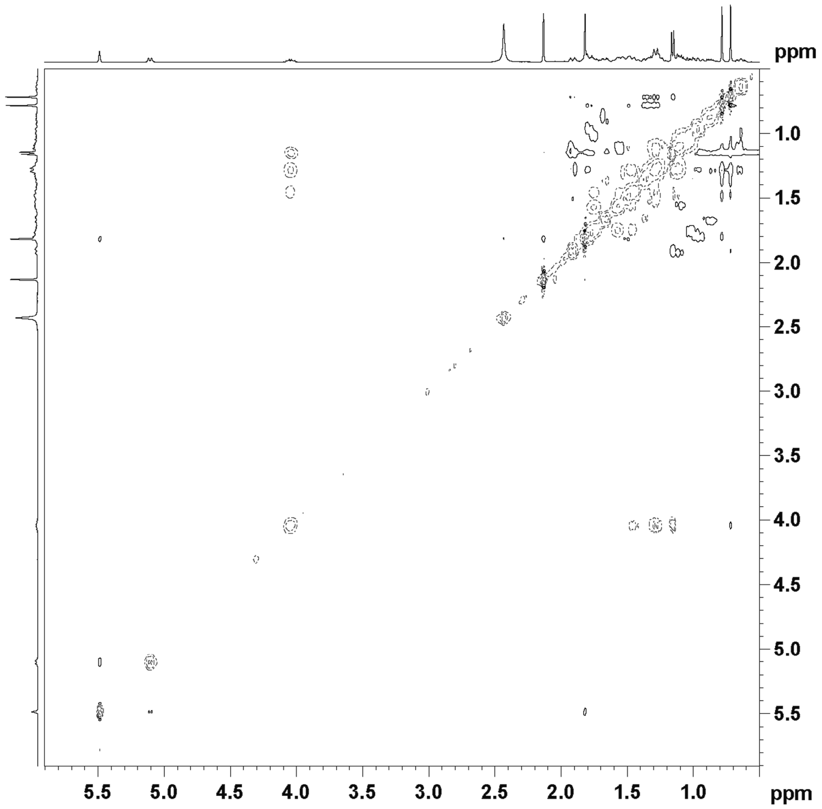

Figure S18. ROESY spectrum of compound **3**.

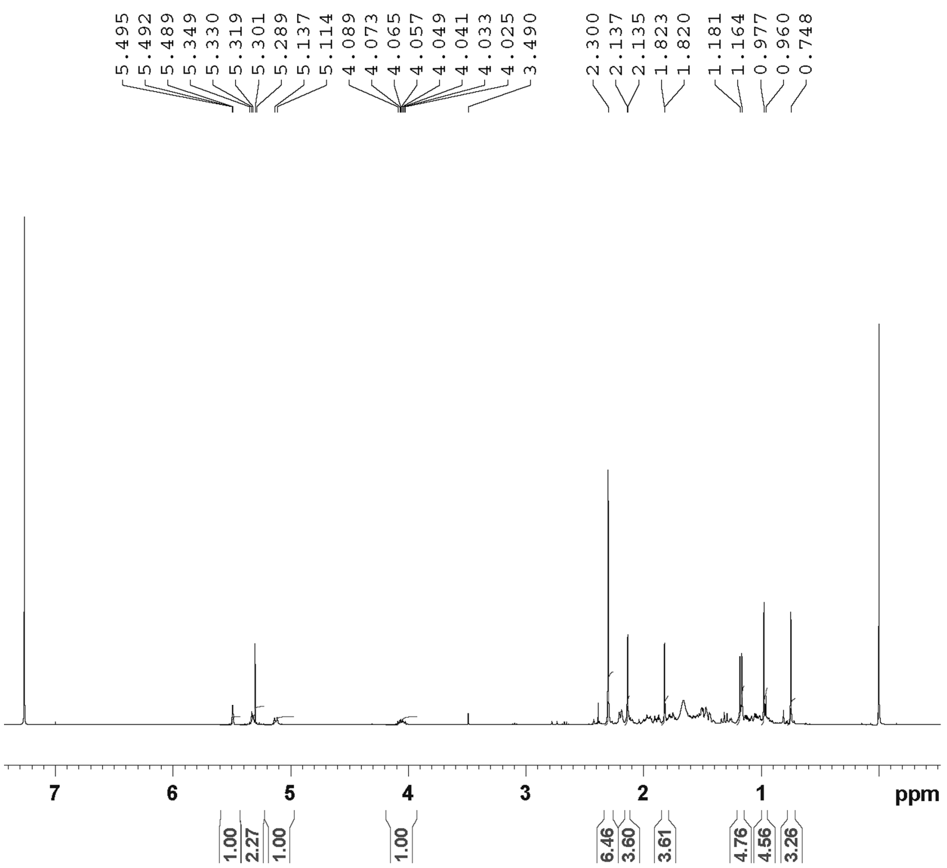

Figure S19. ^{1}H-NMR spectrum of compound **4**.

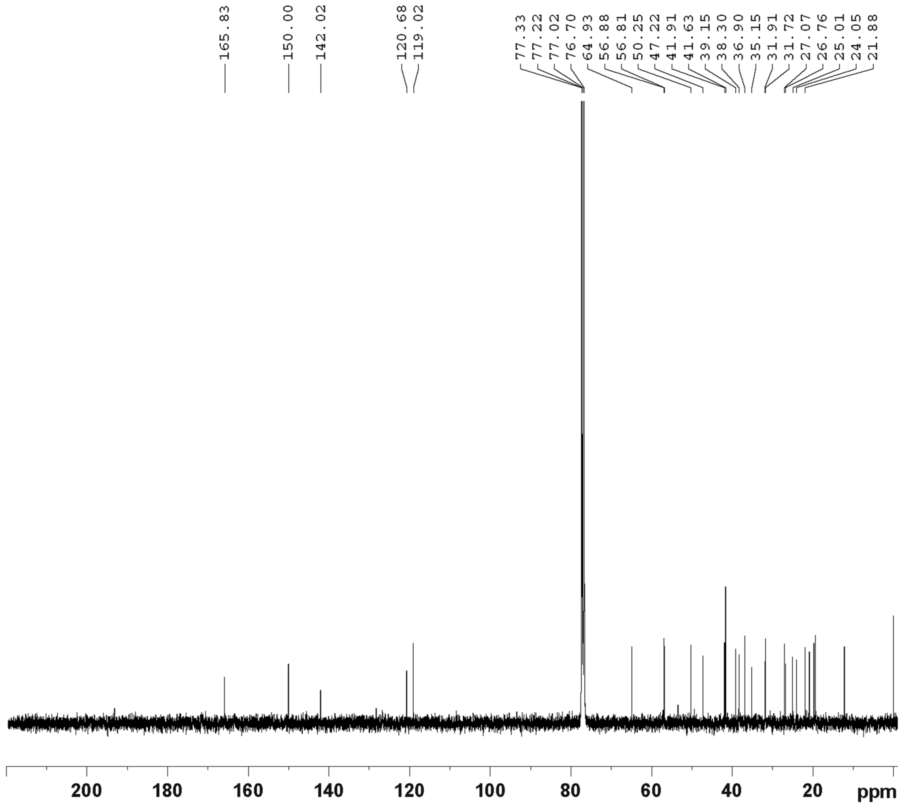

Figure S20. ^{13}C-NMR spectrum of compound **4**.

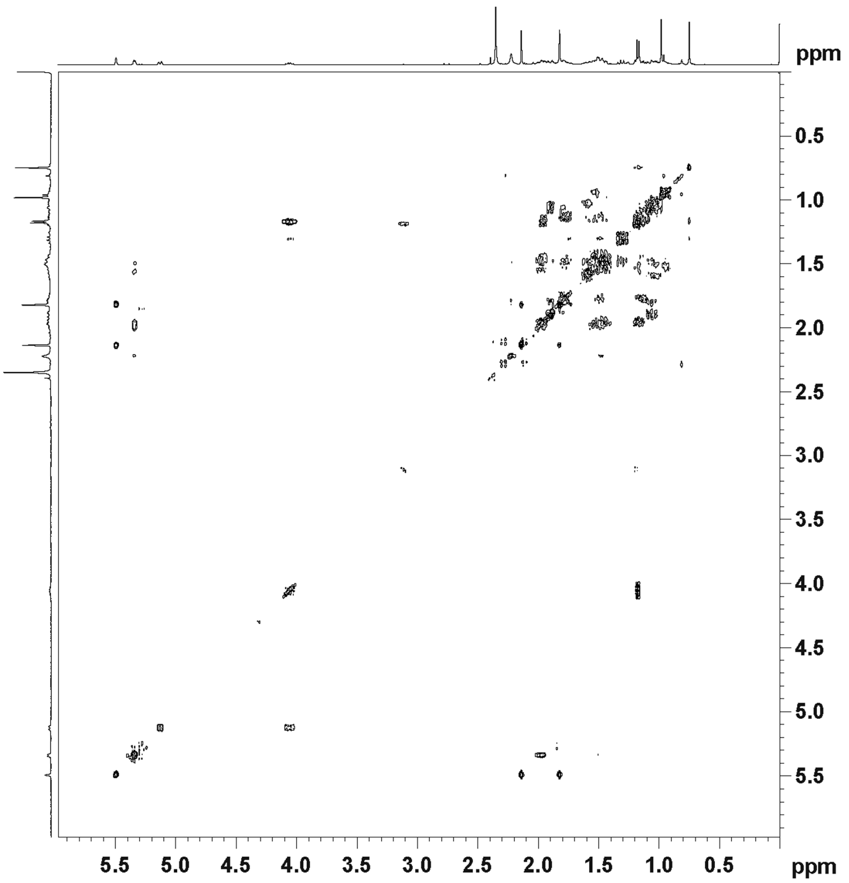

Figure S21. ^{1}H-^{1}H COSY spectrum of compound **4**.

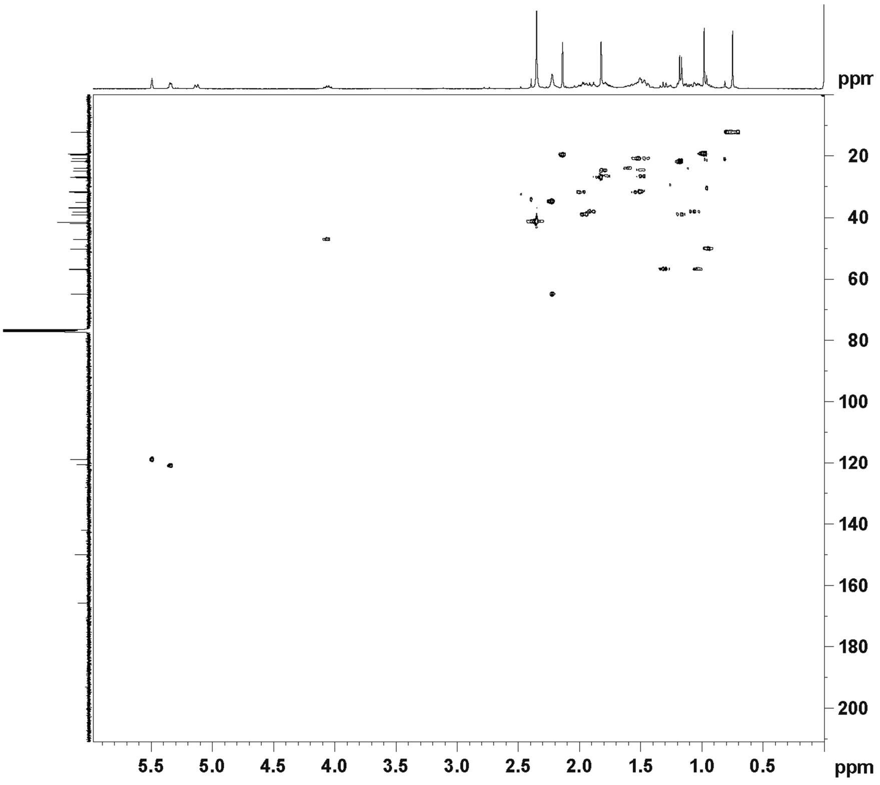

Figure S22. HSQC spectrum of compound **4**.

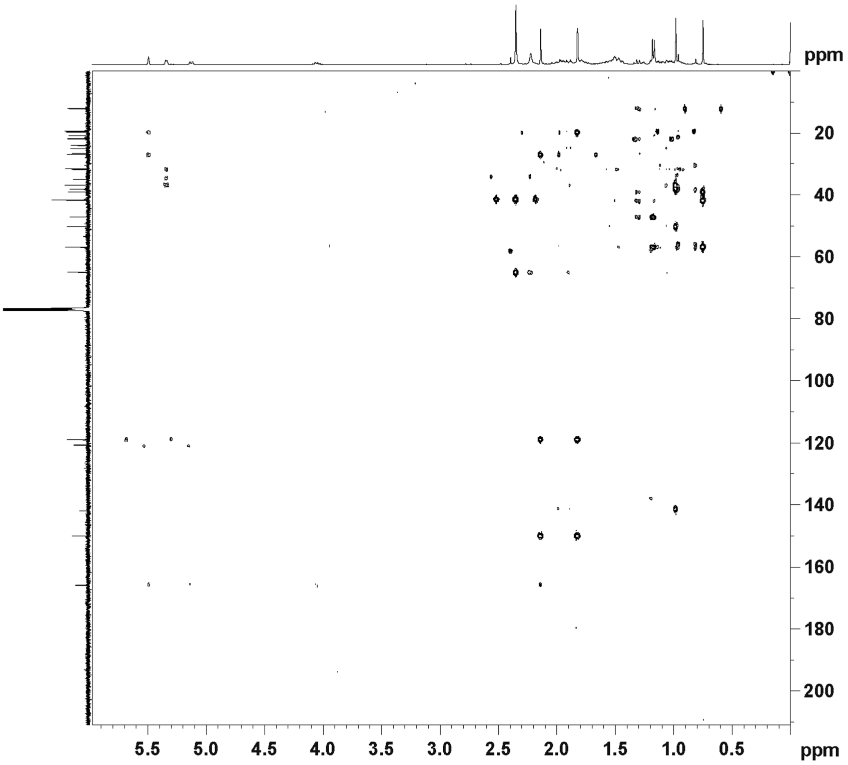

Figure S23. HMBC spectrum of compound **4**.

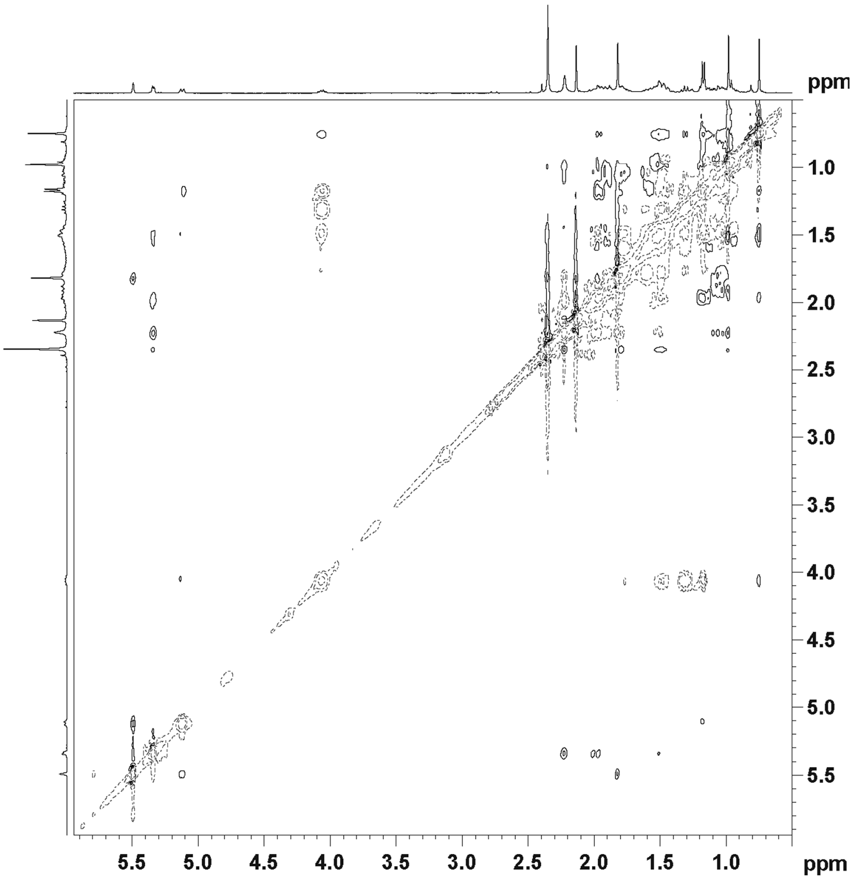

Figure S24. ROESY spectrum of compound **4**.

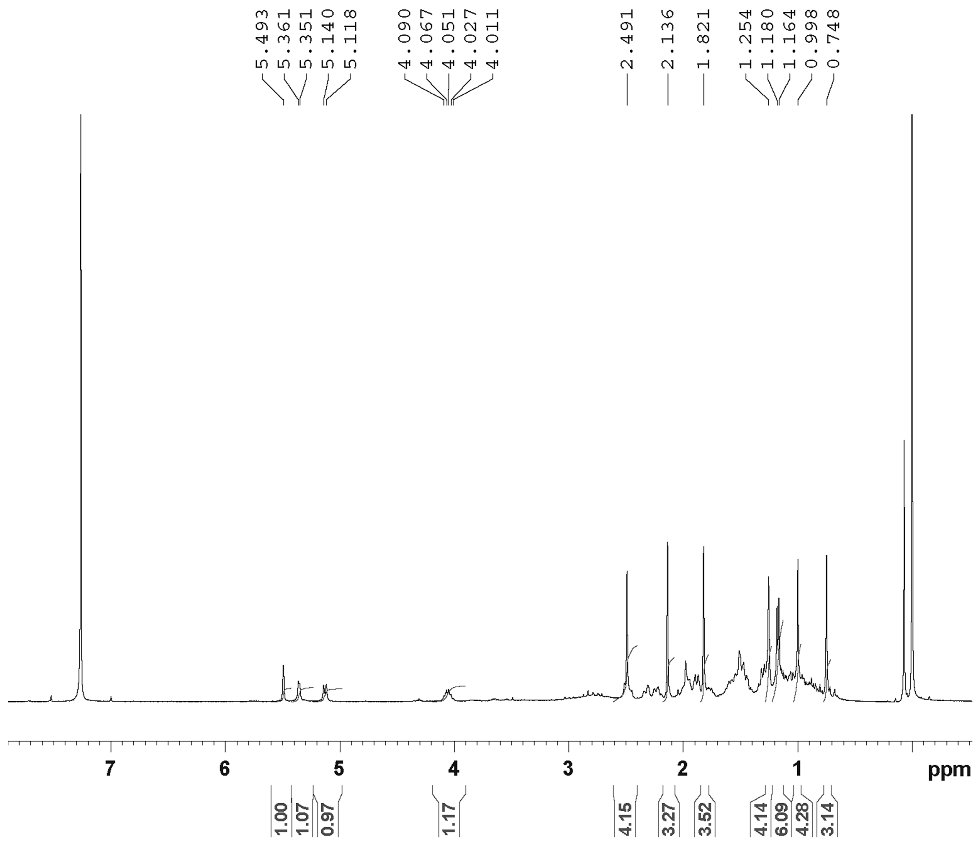

Figure S25. ^{1}H-NMR spectrum of compound **5**.

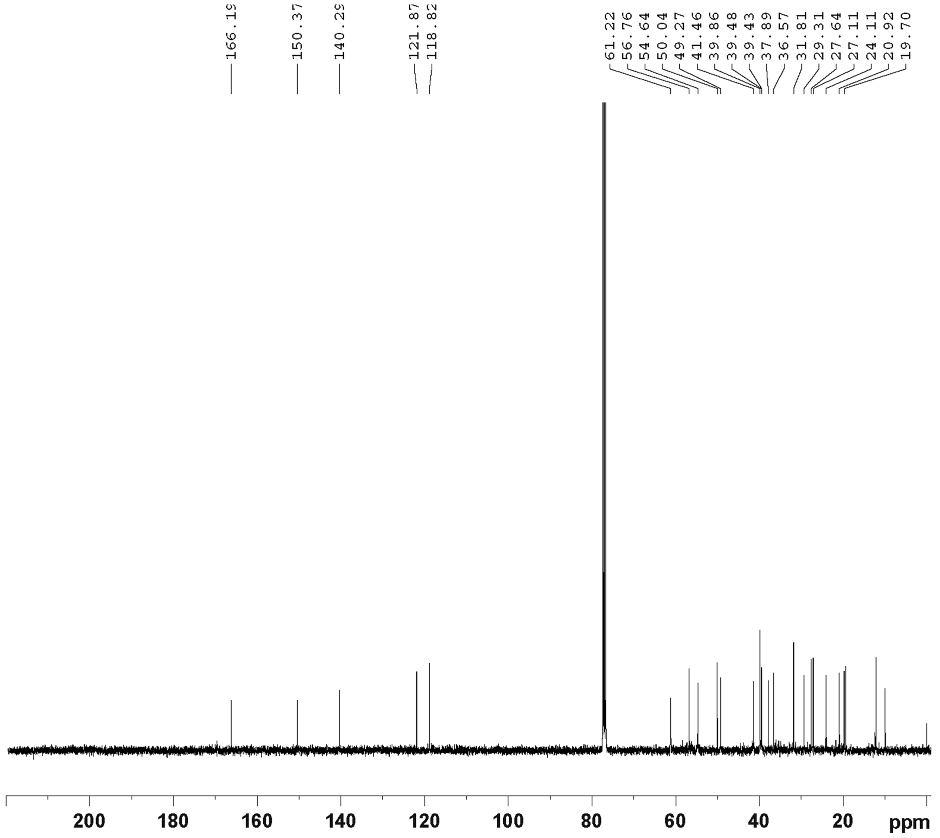

Figure S26. ^{13}C-NMR spectrum of compound **5**.

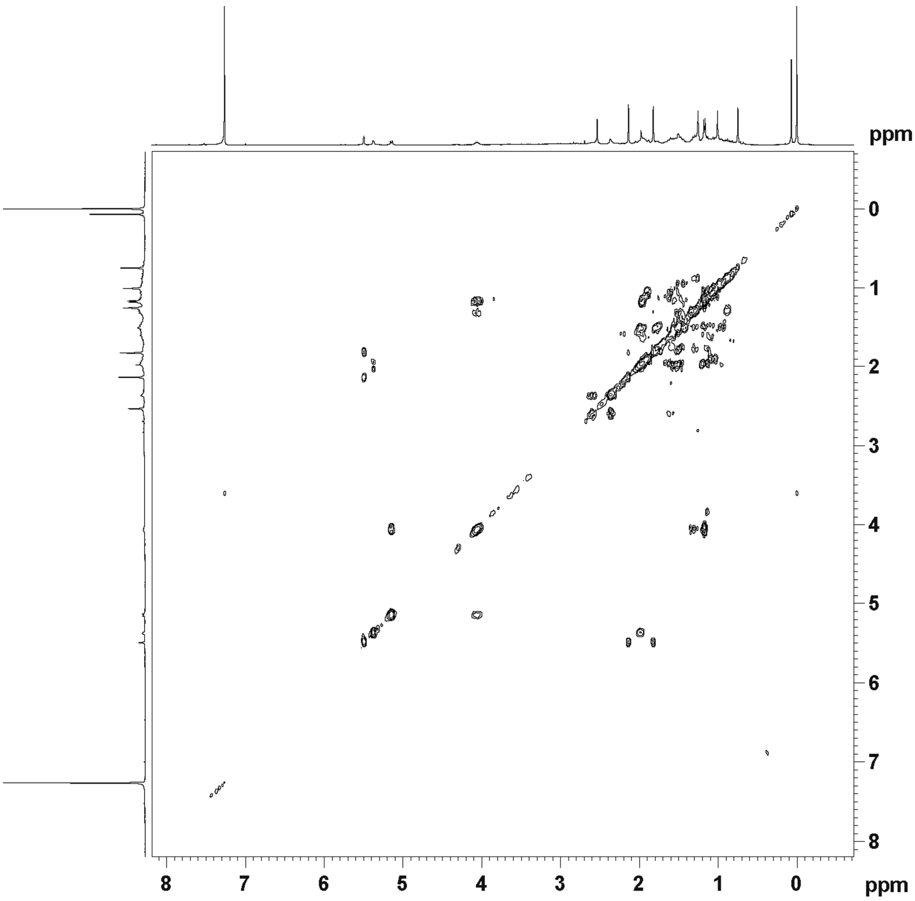

Figure S27. ^{1}H-^{1}H COSY spectrum of compound **5**.

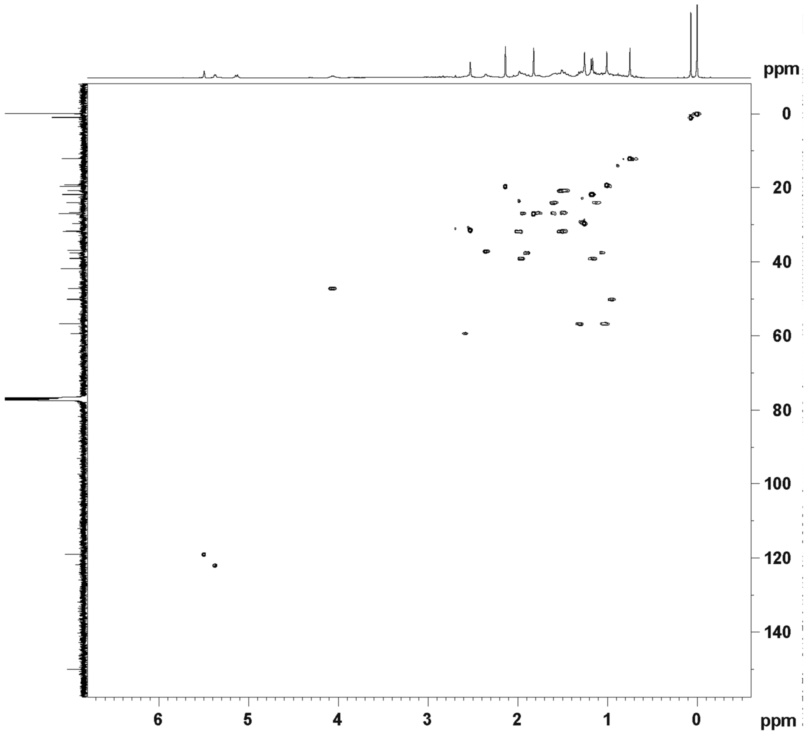

Figure S28. HSQC spectrum of compound **5**.

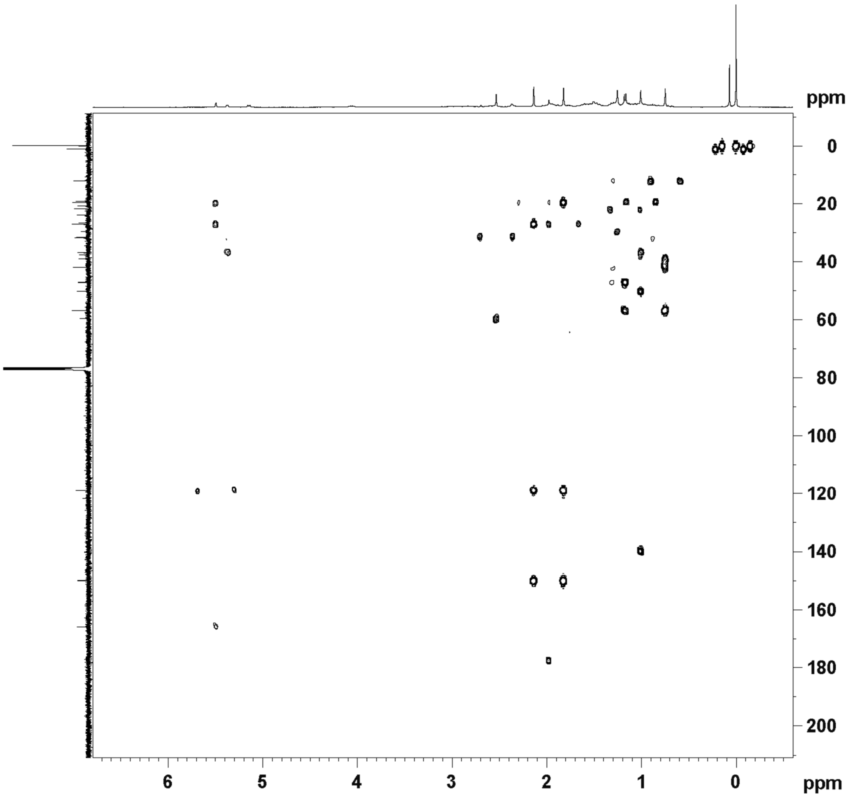

Figure S29. HMBC spectrum of compound **5**.

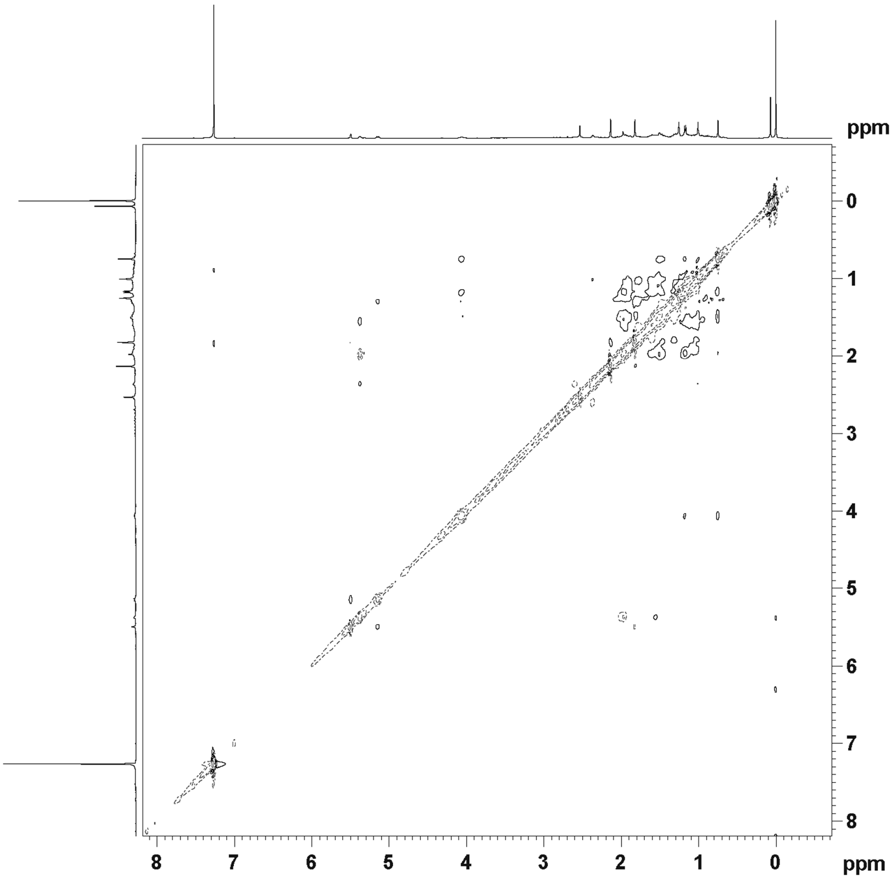

Figure S30. ROESY spectrum of compound **5**.

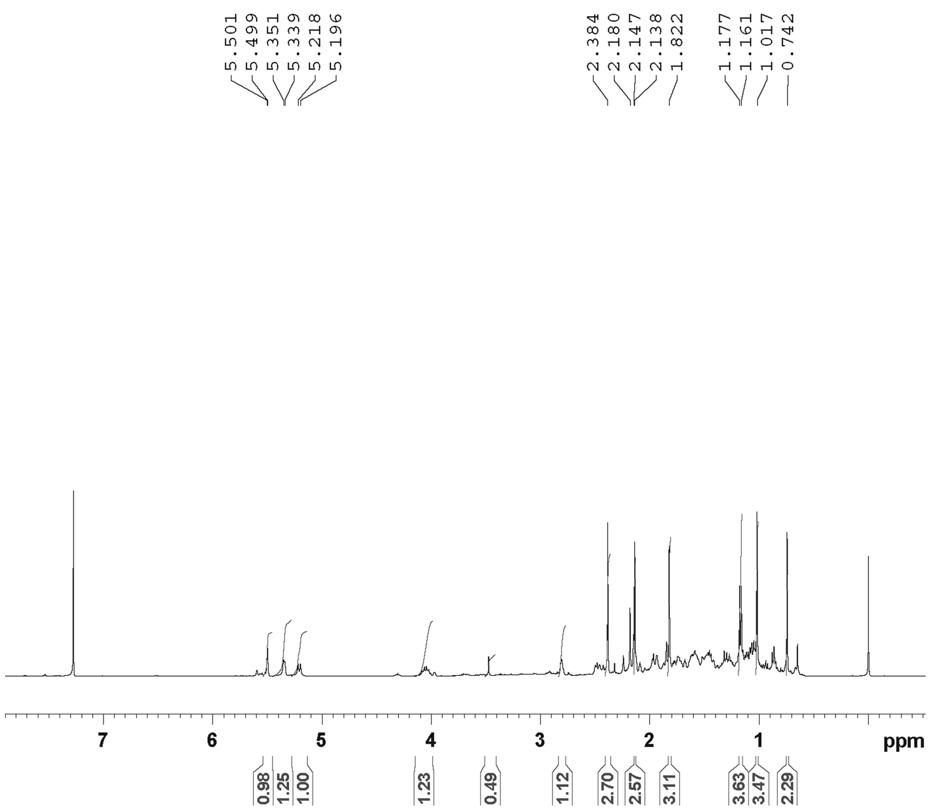

Figure S31. ^{1}H-NMR spectrum of compound **6**.

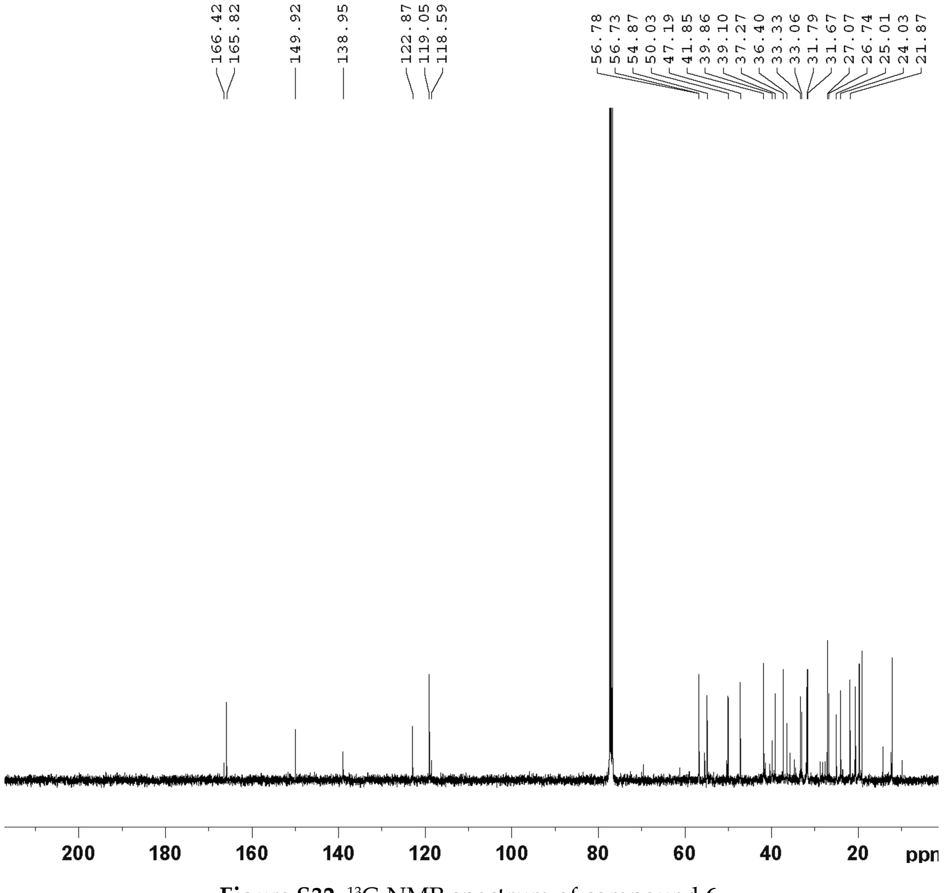

Figure S32. ^{13}C-NMR spectrum of compound **6**.

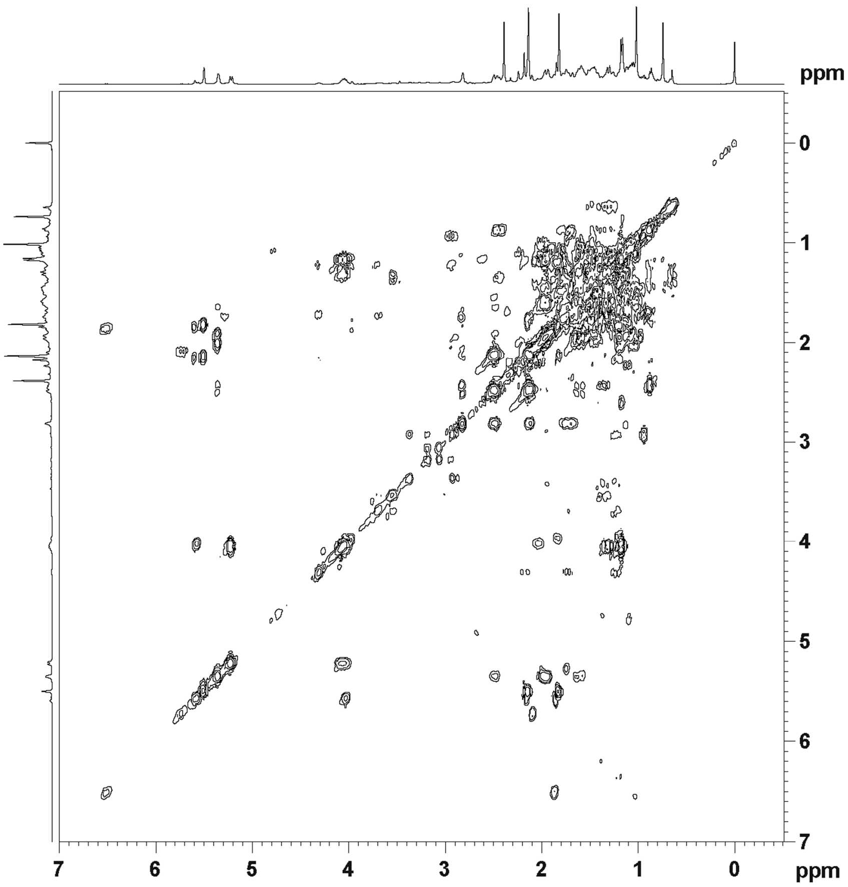

Figure S33. ^{1}H-^{1}H COSY spectrum of compound **6**.

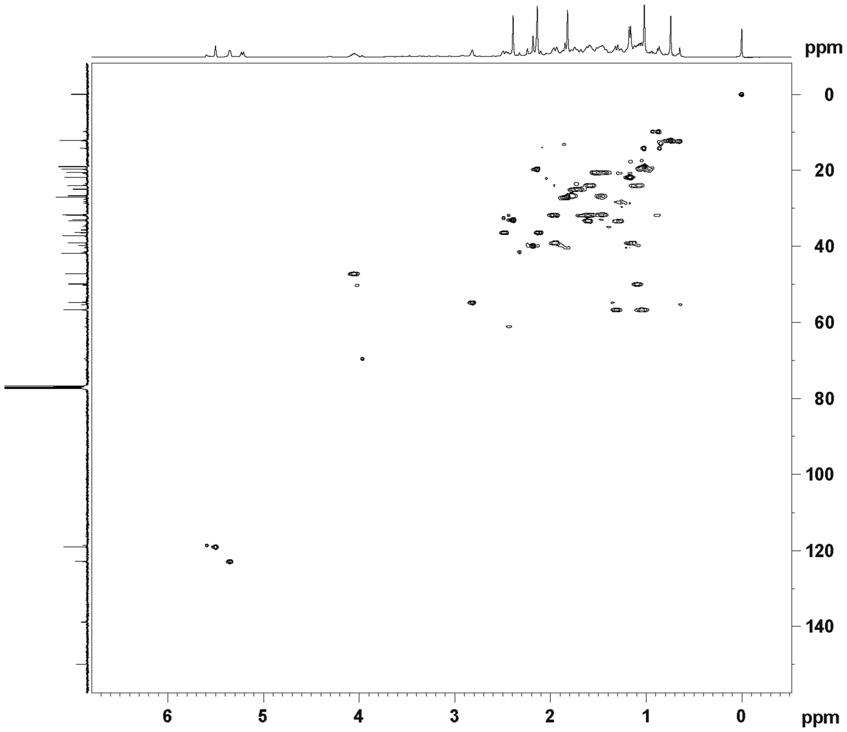

Figure S34. HSQC spectrum of compound **6**.

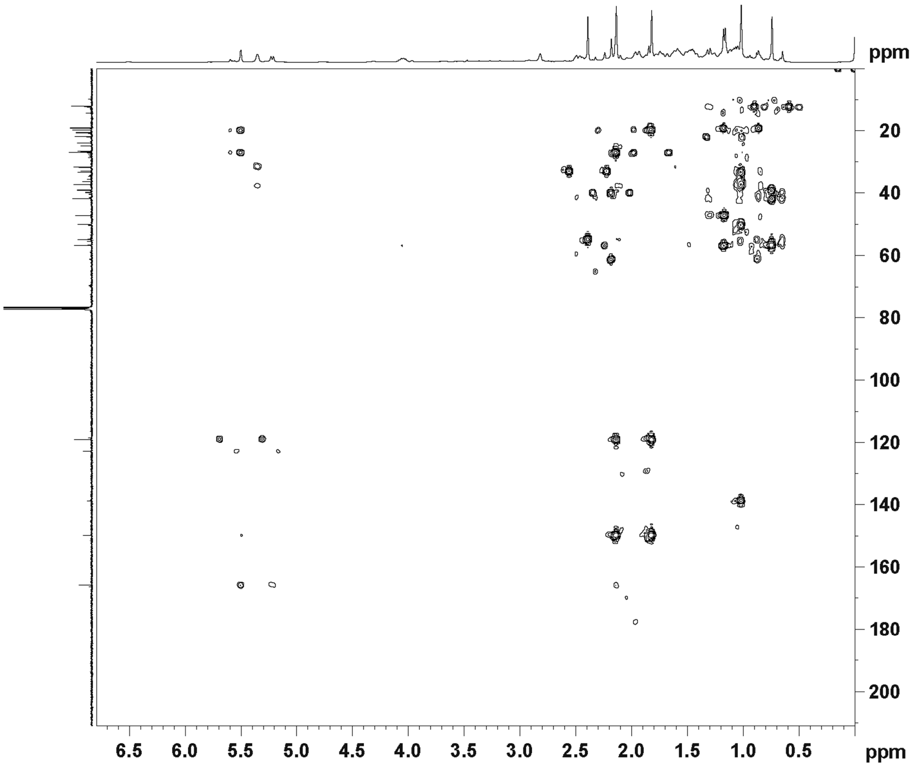

Figure S35. HMBC spectrum of compound **6**.

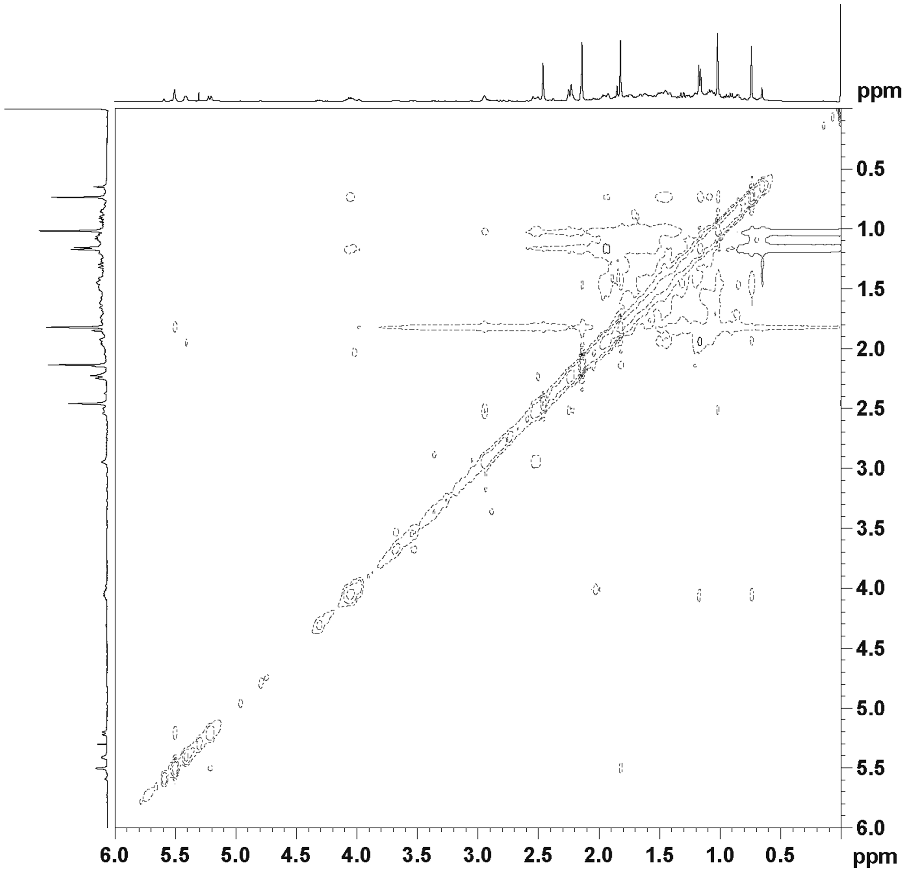

Figure S36. ROESY spectrum of compound **6**.

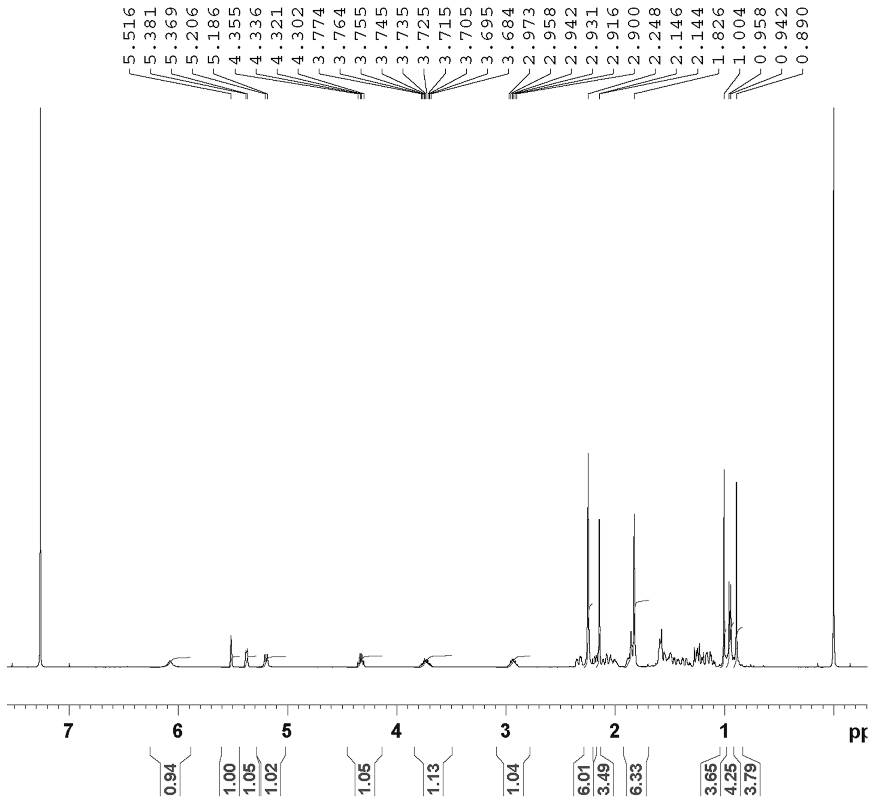

Figure S37. ^{1}H-NMR spectrum of compound **7**.

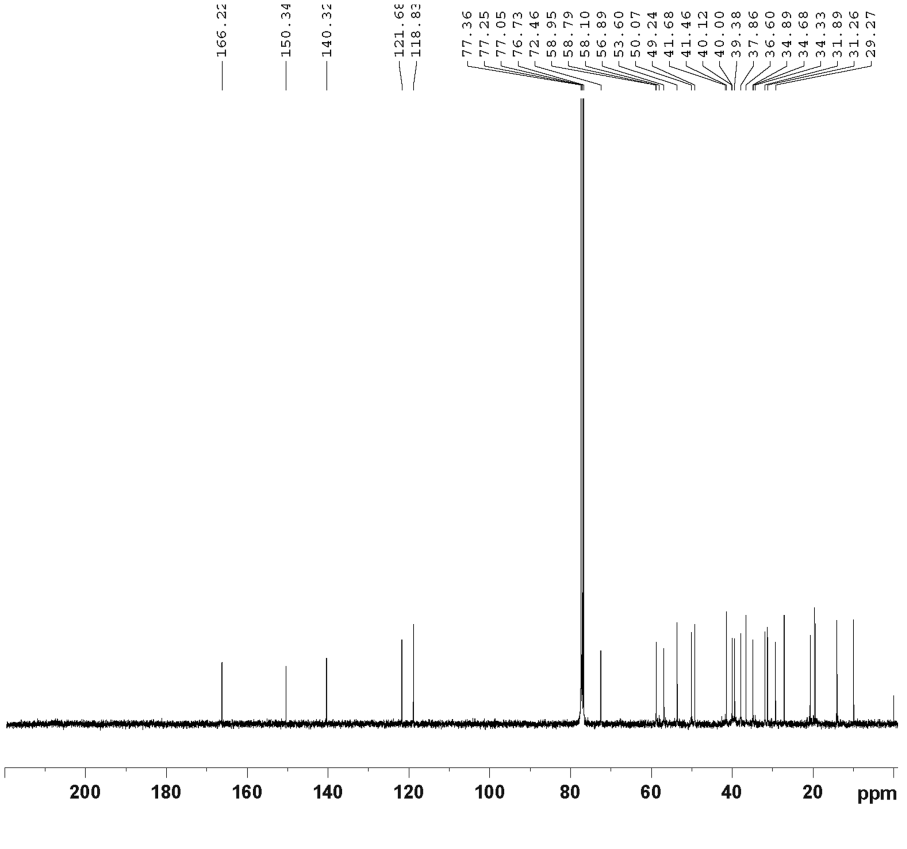

Figure S38. ^{13}C-NMR spectrum of compound **7**.

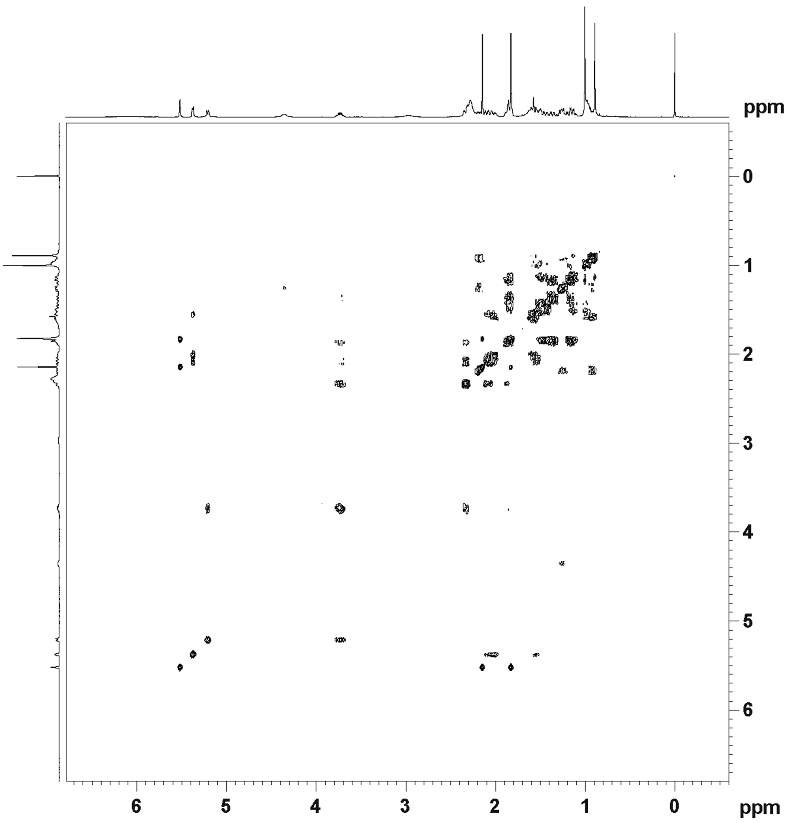

Figure S39. ^{1}H-^{1}H COSY spectrum of compound **7**.

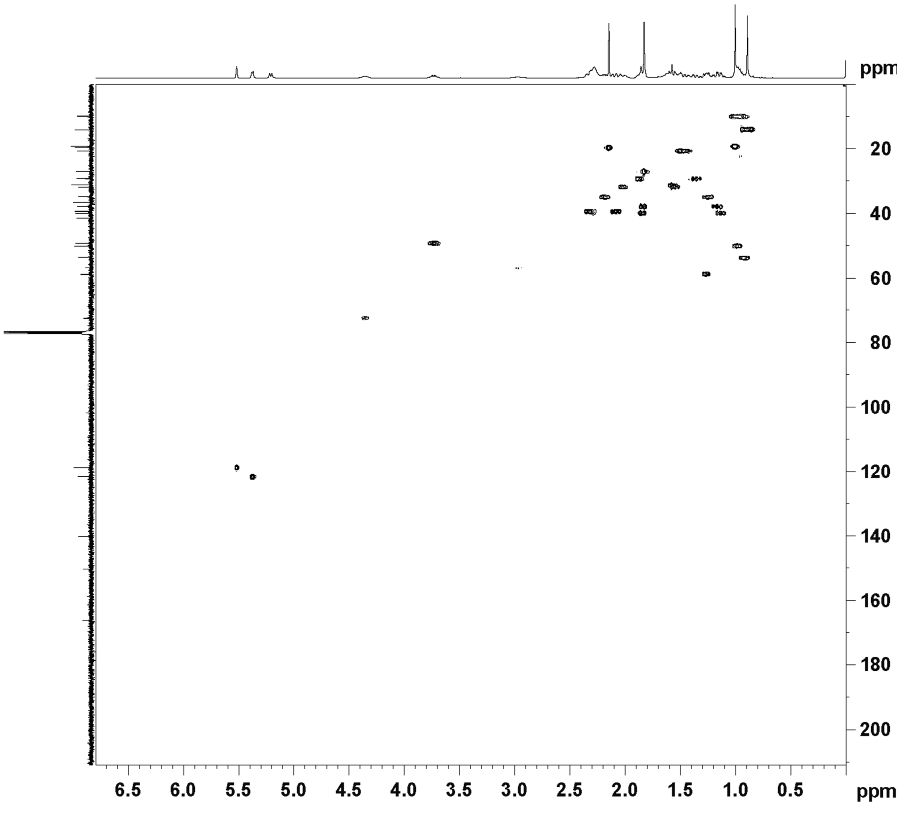

Figure S40. HSQC spectrum of compound **7**.

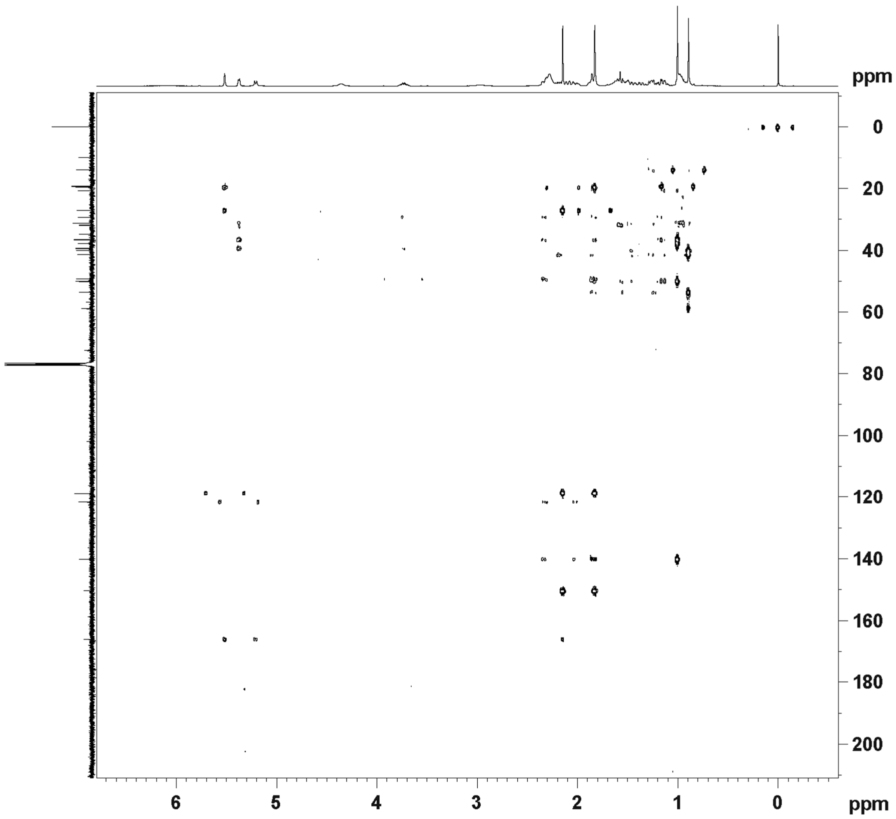

Figure S41. HMBC spectrum of compound **7**.

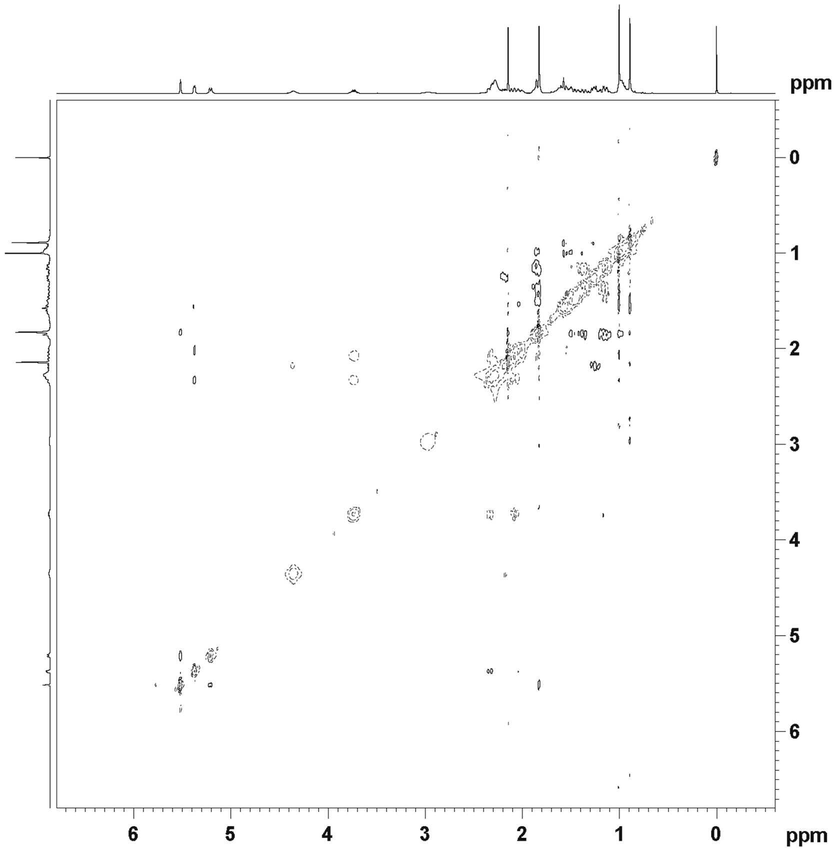

Figure S42. ROESY spectrum of compound **7**.

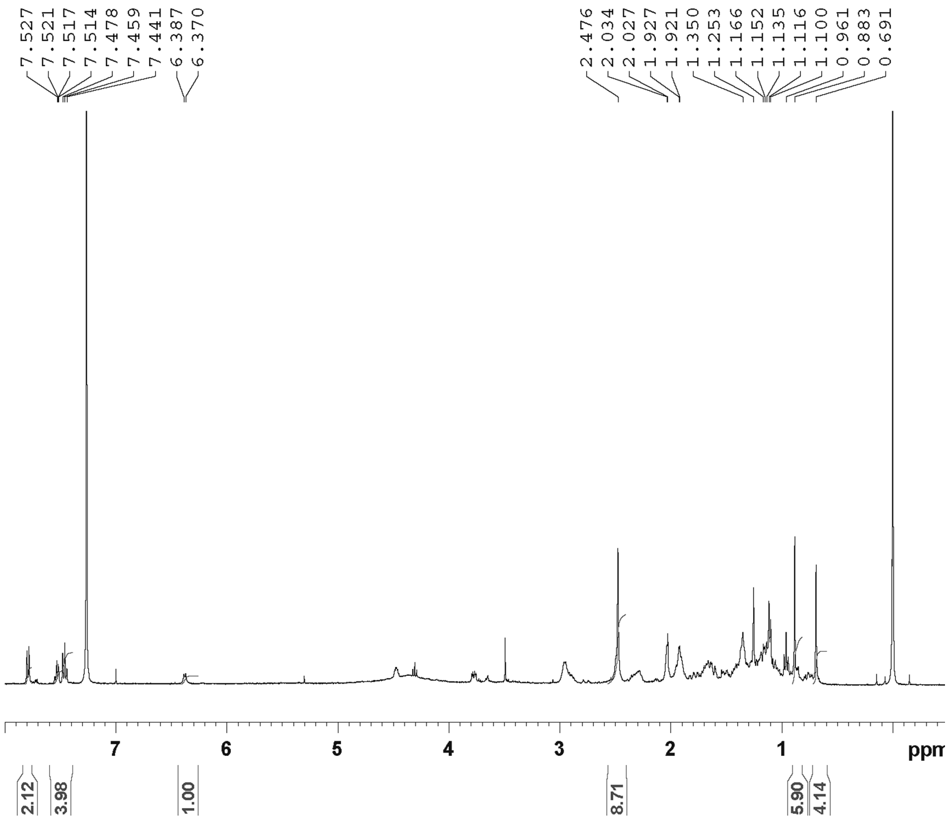

Figure S43. ^{1}H-NMR spectrum of compound **8**.

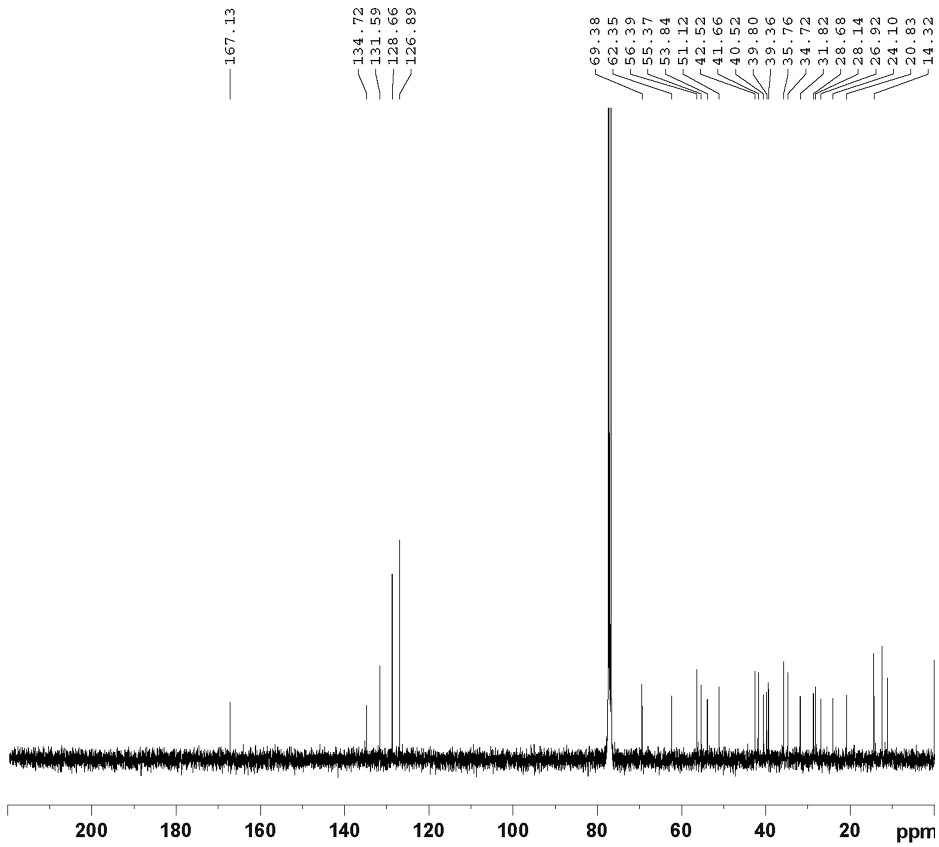

Figure S44. ^{13}C-NMR spectrum of compound **8**.

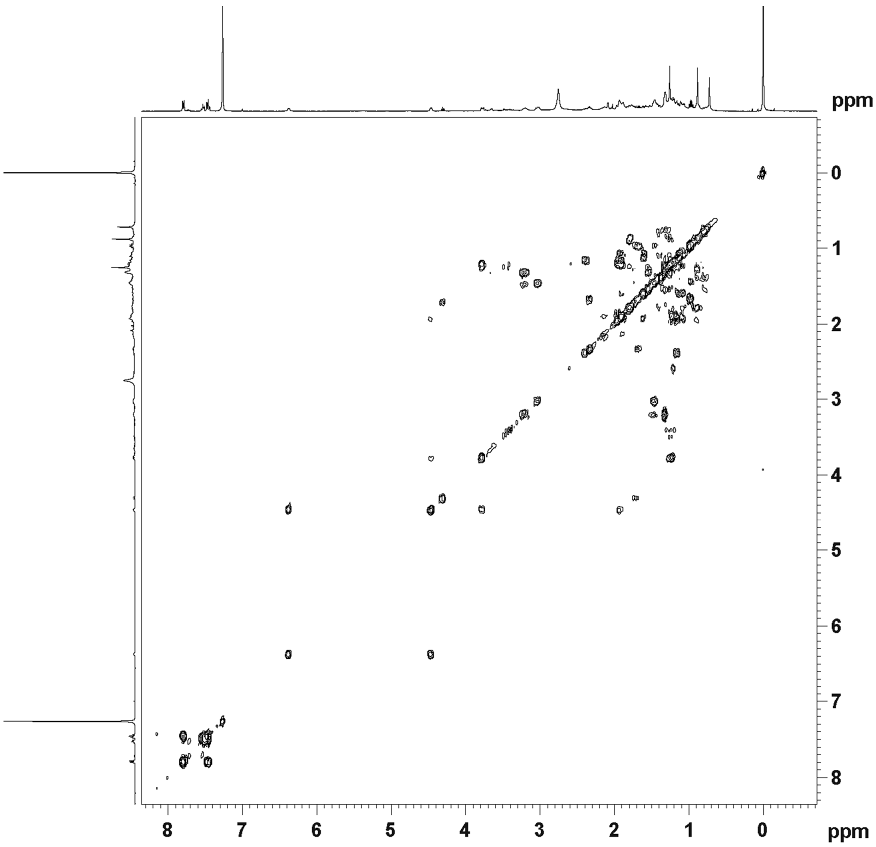

Figure S45. ^1H-^1H COSY spectrum of compound **8**.

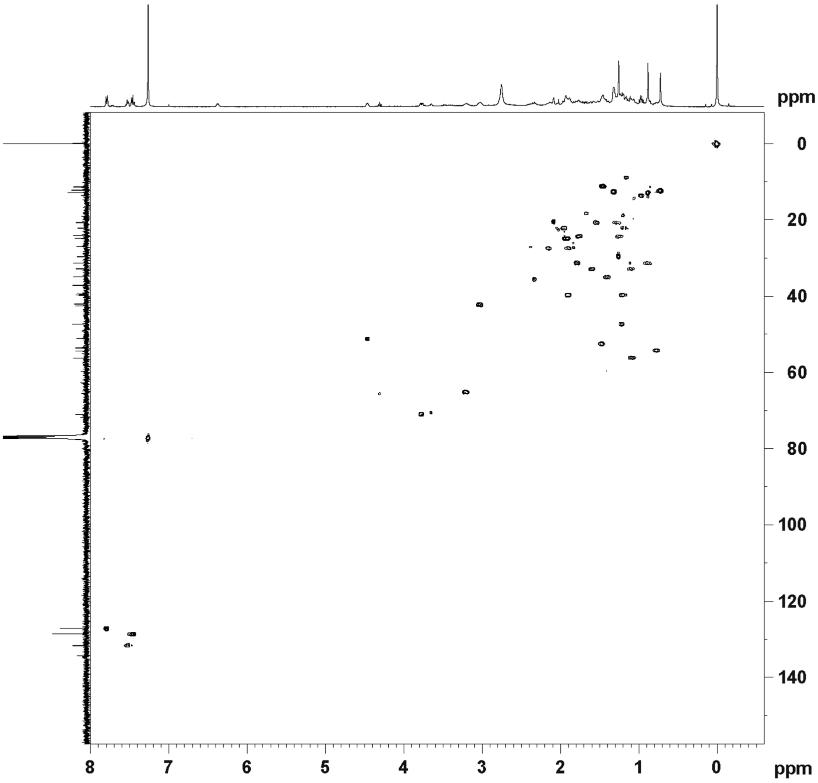

Figure S46. HSQC spectrum of compound **8**.

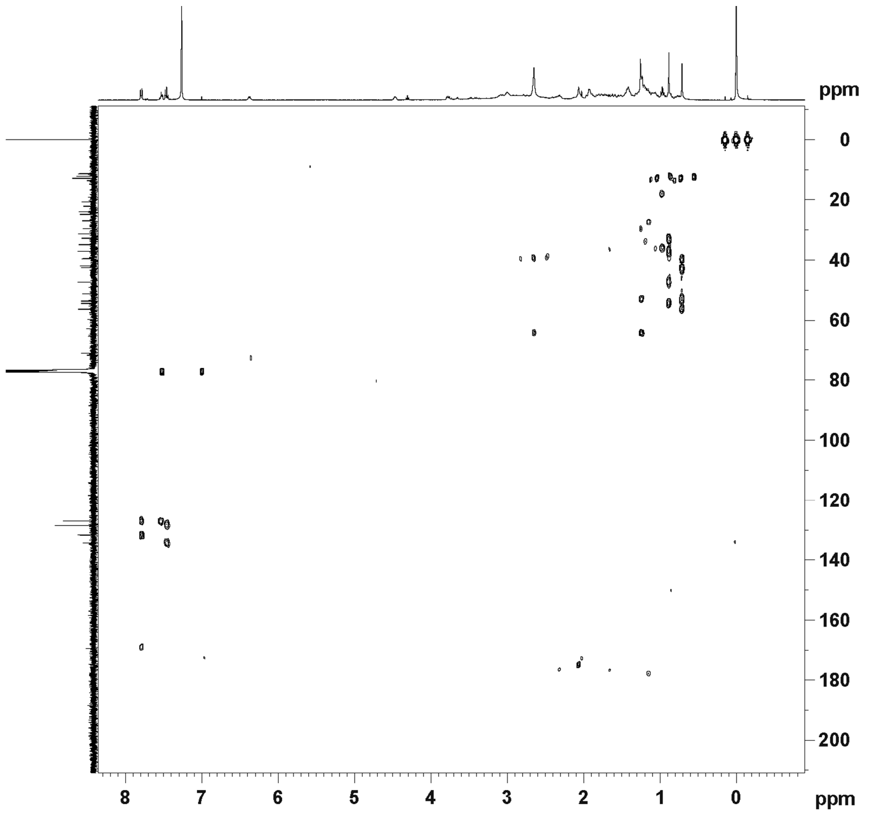

Figure S47. HMBC spectrum of compound **8**.

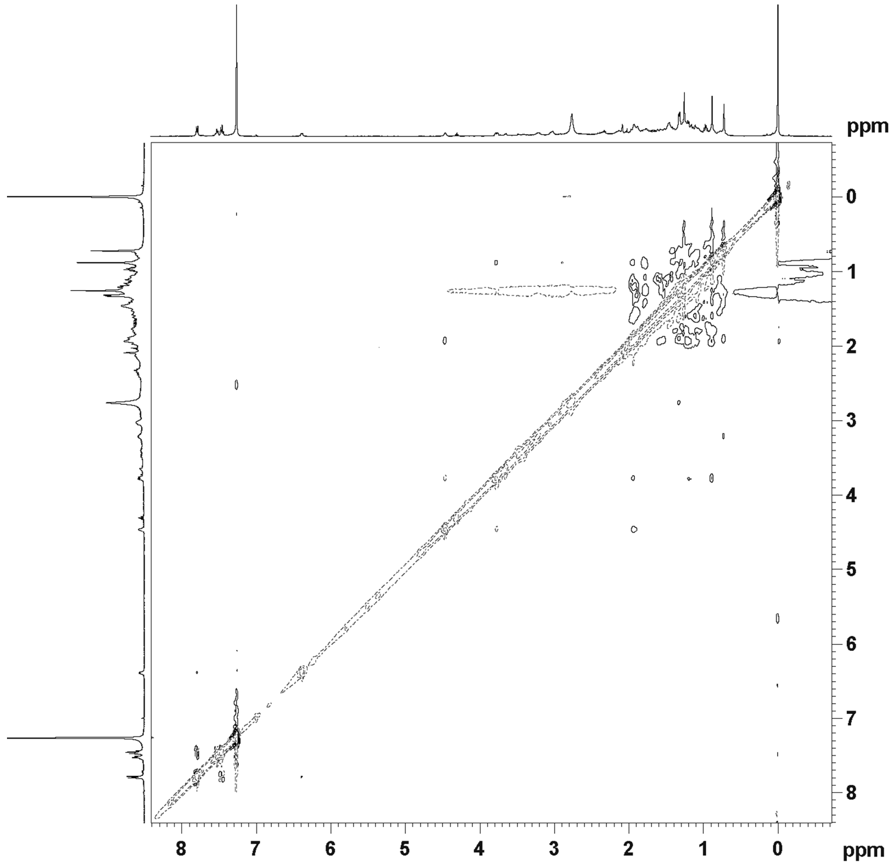

Figure S48. ROESY spectrum of compound **8**.

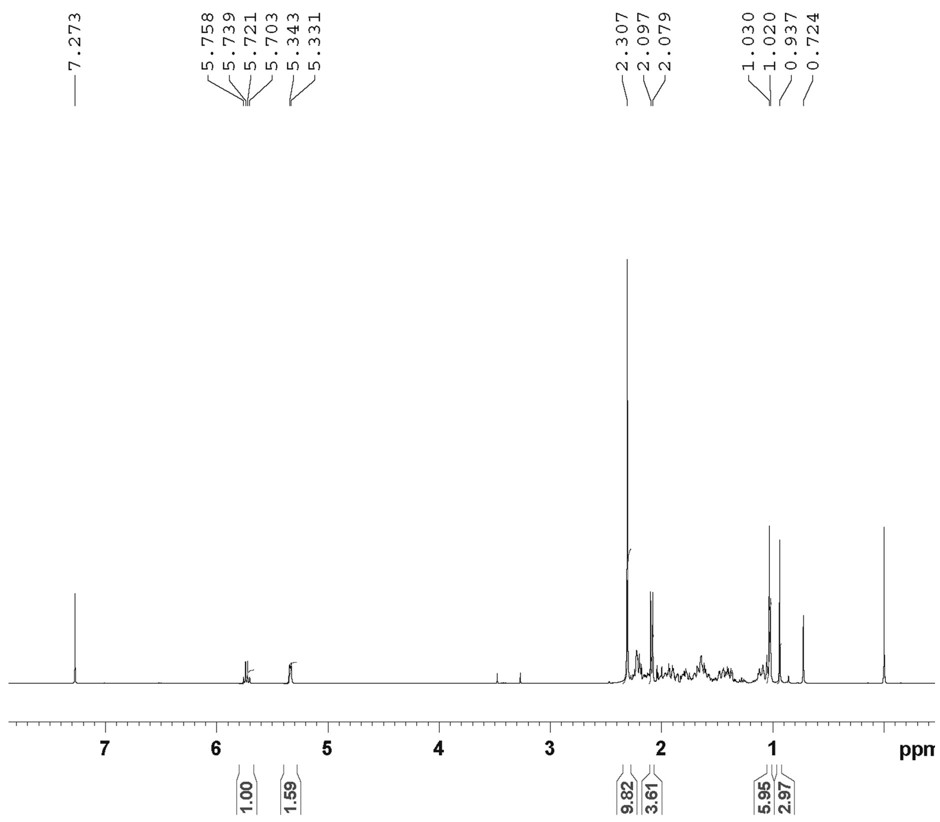

Figure S49. ^{1}H-NMR spectrum of compound **9**.

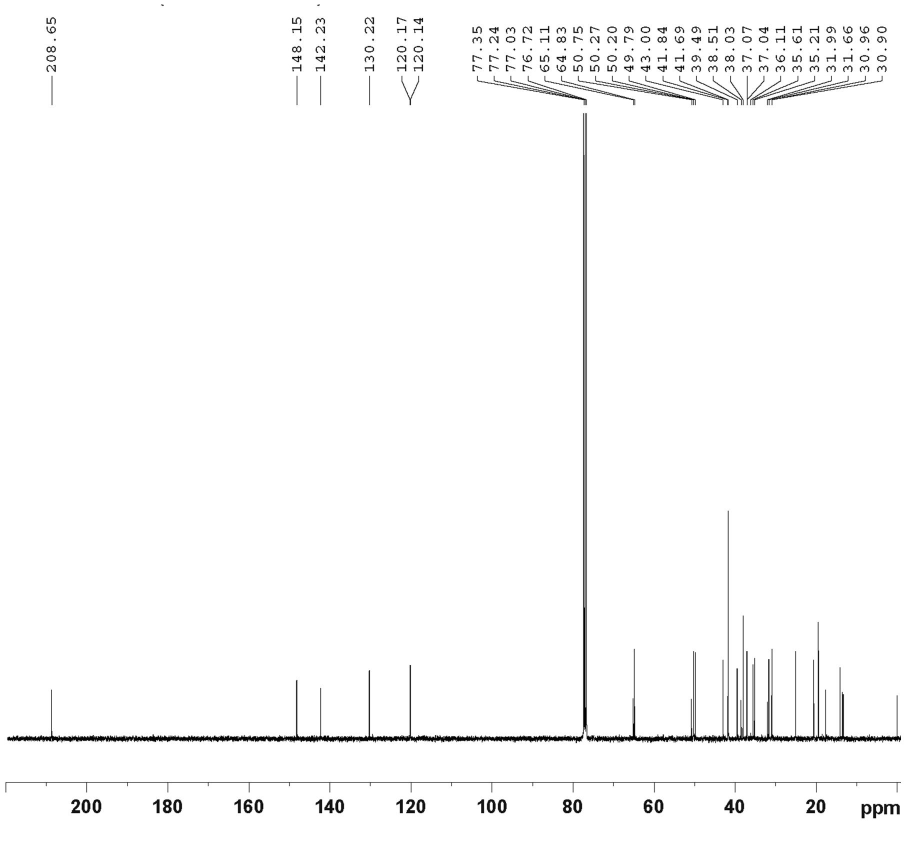

Figure S50. ^{13}C-NMR spectrum of compound **9**.

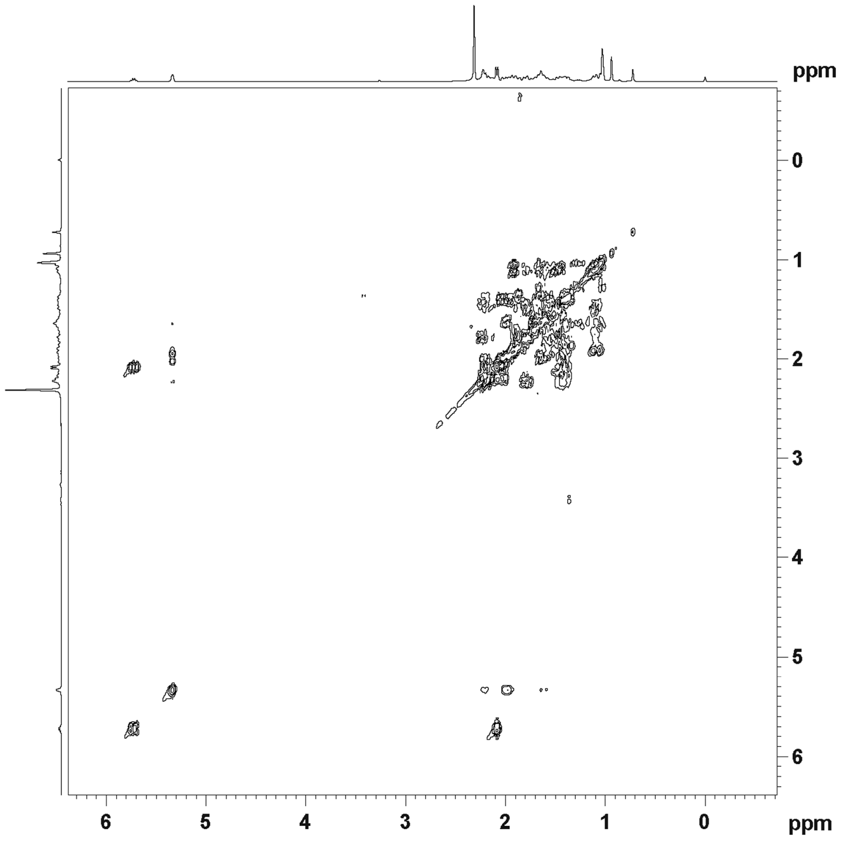

Figure S51. ^1H-^1H COSY spectrum of compound **9**.

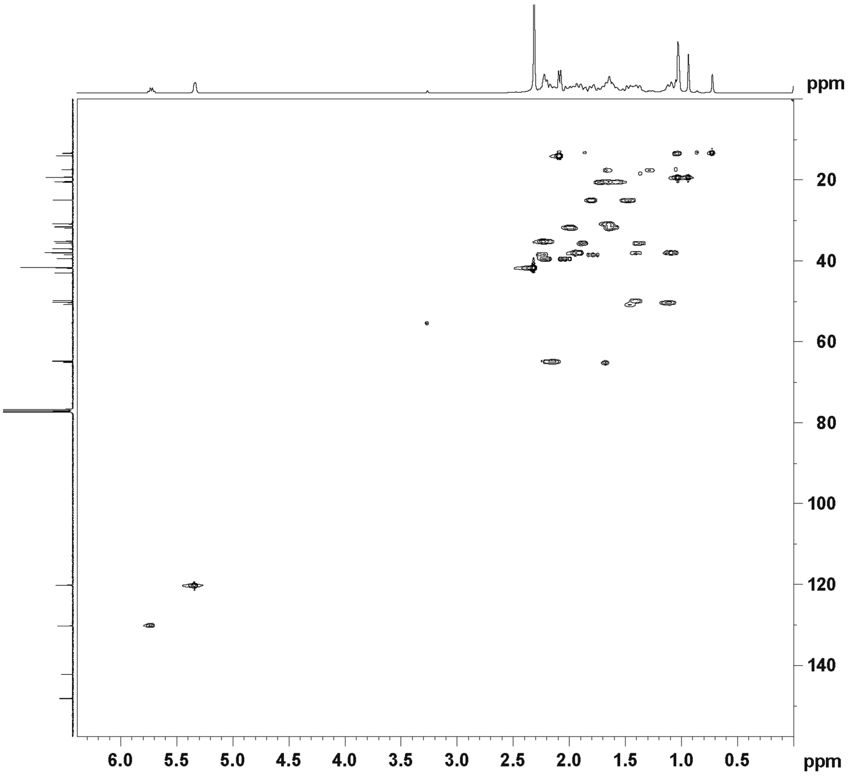

Figure S52. HSQC spectrum of compound **9**.

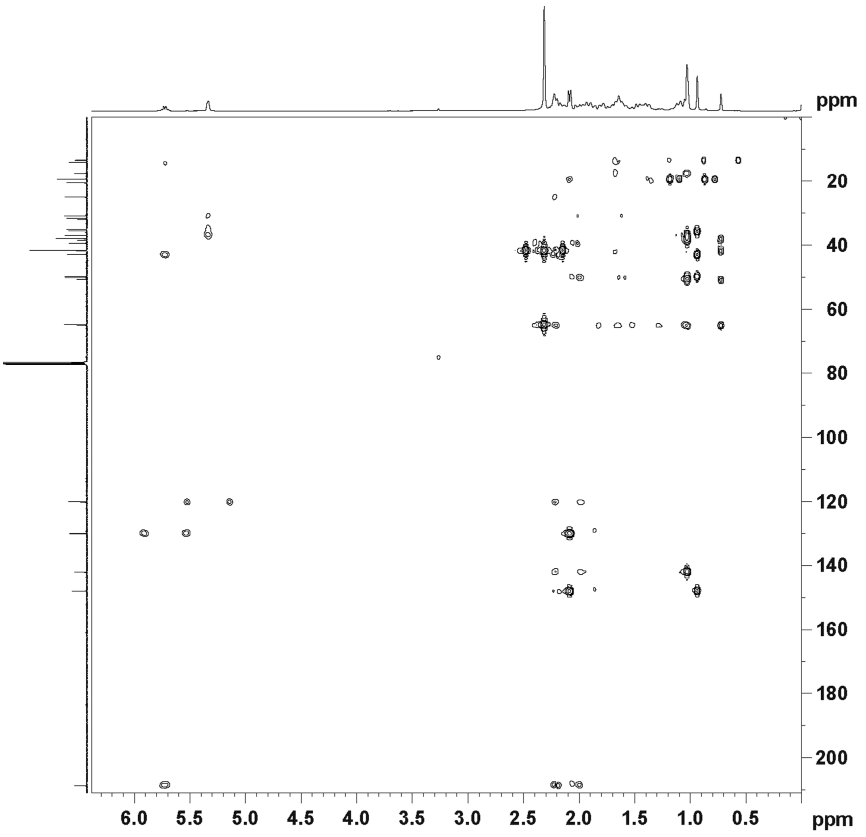

Figure S53. HMBC spectrum of compound **9**.

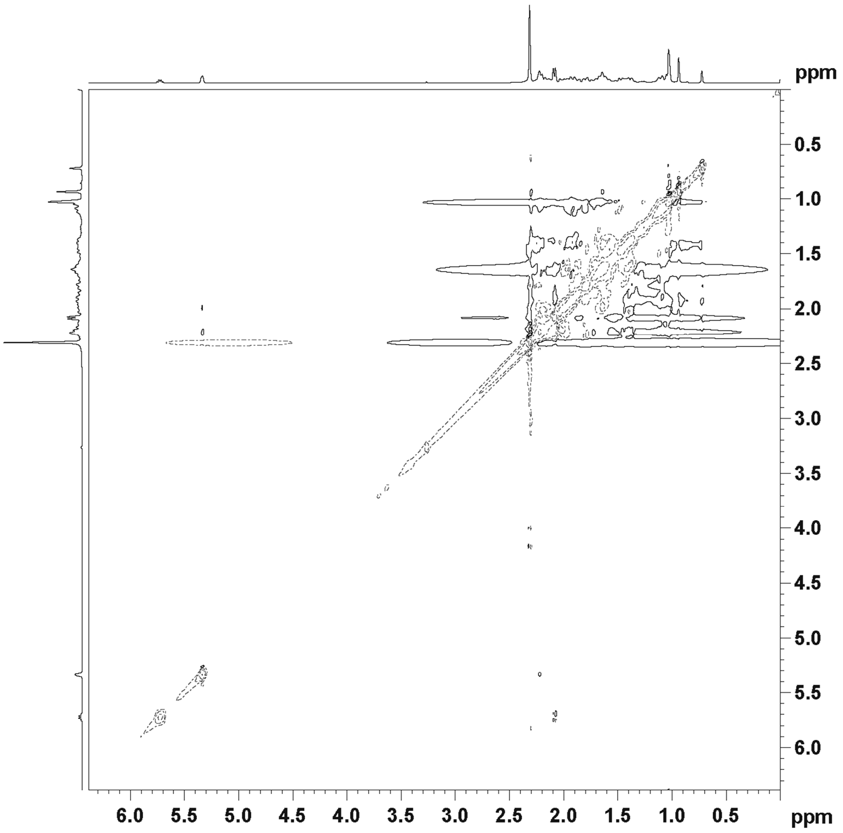

Figure S54. ROESY spectrum of compound **9**.
